# Reactive bromine in volcanic plumes confines the emission temperature and oxidation of magmatic gases at the atmospheric interface

**DOI:** 10.1126/sciadv.adt8607

**Published:** 2025-04-30

**Authors:** Alexander Nies, Tjarda J. Roberts, Guillaume Dayma, Tobias P. Fischer, Jonas Kuhn

**Affiliations:** ^1^ LPC2E, OSUC, University of Orléans, CNRS, CNES, F-45071 Orléans, France.; ^2^ LMD/IPSL, ENS, Université PSL, École Polytechnique, Institute Polytechnique de Paris, Sorbonne Université, CNRS, F-75005 Paris, France.; ^3^ ICARE, University of Orléans, CNRS, F-45071 Orléans, France.; ^4^ Earth and Planetary Sciences Department, University of New Mexico, Albuquerque, NM 87131, USA.; ^5^ Department of Atmospheric and Oceanic Sciences, University of California, Los Angeles, CA 90095, USA.

## Abstract

The redox composition of volcanic gases relays substantial information about magmatic conditions and volcanic activity. Volcanic plume gas measurements are often interpreted assuming that magmatic gases are chemically inert on emission and near-source dilution in air. Conversely, many volcanic plumes contain high levels of bromine monoxide (BrO), which is produced by atmospheric oxidation of magmatic hydrogen bromide (HBr) emissions. We investigate the chemical kinetics of hot magmatic gases mixing with air. Our model reproduces and explains observations of volcanic plume BrO at Mt. Etna, evidencing that reduced gases [HBr, as well as carbon monoxide (CO) and hydrogen (H_2_)] can oxidize at the hot magma–air interface. The extent of oxidation is controlled by the magmatic gas temperature. Observations of BrO and H_2_ in Mt. Etna plume indicate that magmatic gases enter air at several hundred kelvin below magmatic temperature, consistent with hypothesized decoupling of gas temperatures from magma prior to emission.

## Teaser

When volcanoes release hot gases into air, fast chemical reactions occur, which need to be traced to understand magma conditions.

## INTRODUCTION

A leading question in volcanic gas geochemistry is to what extent a gas sample represents the composition of the magmatic gas, hence reflects conditions inside the volcano. The magmatic gas chemistry depends on the composition and temperature of the magma from which it exsolves and is altered by magma dynamics. Furthermore, magmatic gases can interact with water, rock, or atmospheric air (*1*, *2*). Avoiding all of these interactions during sampling is a challenging task and can only be achieved with great effort at a few specific locations [high-temperature fumaroles or lava tube outlets, e.g., (*3*–*6*)]. In contrast, it is much easier to perform measurements of the composition of volcanic plumes, i.e., magmatic gases diluted in air. However, using volcanic plume gas measurements to trace magma evolution and the activity status of the volcano requires a profound quantitative understanding of the chemical processes inside volcanic plumes, including those happening on short seconds-minutes timescales between gas emission and measurement.

As magma cools or depressurizes, volatile species dissolved in magma exsolve and form a separate fluid phase that either is released alongside the melt (closed system, e.g., fire fountain event) or can migrate some distance through the magma in bubbles (open system, e.g., continuous passive degassing). Volatiles have pressure-dependent solubilities, so the composition of exsolved gases changes with pressure during ascent. Exsolved fluids can further change in their composition by chemical interaction with water or host rocks, before being released to the atmosphere, where they are very rapidly dilute in air. These processes vary as a function of the volcanic environment and activity. Thus, field measurements of volcanic gases can be valuable tracers of volcanic activity for eruption hazard monitoring. Plume parameters such as SO_2_ fluxes and CO_2_/SO_2_ ratios are often used for monitoring purposes as these gases can be considered chemically inert on minute to hour timescales in the atmosphere. The composition of volcanic gas emissions also includes halogens (emitted in reduced form, e.g., HBr and HCl) and other reduced gases (e.g., H_2_, CO, and H_2_S), which carry valuable complementary information on volcanic activity and magmatic conditions [e.g., (*7*)]. Measurements of reduced gases are commonly expressed as redox pairs (e.g., H_2_/H_2_O, CO/CO_2_, and H_2_S/SO_2_) with the composition frequently interpreted as directly reflecting the magmatic redox and temperature conditions [see, e.g., (*8*)]. However, this link between magma degassing and plume gas composition can be perturbed or even destroyed by processes between gas, magma, and rock. An area of long-standing and ongoing debate is the extent that magmatic gas compositions are buffered by melt or mineral interactions or by a sulfur gas buffer en route to emission [see (*9*–*12*)]. The potential for decoupling of magmatic and gas temperatures through adiabatic expansion and cooling of rising gas bubbles has also recently been inferred from discrepancies between observed plume gas composition, magma redox composition calculations, and petrological measurements (*13*, *14*).

The potential influence of these subsurface processes on modifying gas composition is included in efforts to relate observations of volcanic plume gas compositions to magmatic redox state (*11*). However, the effect of atmospheric processing is typically ignored under the assumption that magmatic gases are quenched on emission to air (*15*). This assumption must be scrutinized against available field studies and state-of-the-art high-temperature chemical kinetics. Gas reactions, their rates, and temperature dependencies are quantified through extensive laboratory experiments and high-level ab initio calculations, yielding rate equations that are incorporated into reference databases, which underpin all atmospheric chemistry and combustion chemistry models (*16*–*18*). In cooled volcanic plumes, theory and observations are consistent in reporting slow and negligible oxidation of reduced gases (H_2_, CO, and H_2_S) over plume ages on the order of hours [see, e.g., (*19*)]. In high-temperature mixtures of magmatic gases with air, theory predicts substantial oxidation of reduced gases (*20*–*22*), disproving earlier hypotheses that these gases might be treated as inert (*23*). Current research explores the potential for high-temperature reactions in magmatic gas-air mixtures to lead to decoupling of plume gas composition from the emitted composition and magmatic redox state (*20*). Observational evidence is mixed: An absence of H_2_ in some observed plume compositions suggests fast chemical oxidation in air [e.g., (*24*, *25*)], whereas other field observations report compositions that demonstrate that reduced gases are preserved in the plume [e.g., (*26*)]. Characterizing the very early plume chemistry through field measurements on second-scale plume ages is extremely challenging [see, e.g., (*27*)].

The oxidative nature of volcanic plumes is nevertheless clearly demonstrated for bromine. It is well established that BrO is formed by chemical reactions in volcanic plumes as evidenced by field observations [e.g., (*28*–*31*)]. Atmospheric chemistry models have been used to simulate the oxidation of emitted volcanic HBr into BrO via the heterogeneous autocatalytic “bromine explosion” mechanism [e.g., (*32*–*35*)]. However, models could only reproduce observed BrO levels when a representation of the chemistry of the early-stage high-temperature plume was assumed in their initialization. These studies have all relied on treating the early plume as an high-temperature reactor in thermochemical equilibrium [e.g., (*36*, *37*)]. Such thermochemical equilibrium calculations on mixtures of hot magmatic gases with air predict the presence of radical species like OH and reactive halogen species like Br atoms. Including these radicals in the initialization of atmospheric chemistry models was found to promote halogen oxidation, improving the agreement between modeled and observed volcanic plume BrO (*32*, *34*, *35*, *38*). However, despite its ubiquitous use in volcanology because the approach was introduced in (*39*) to volcano geochemistry and in (*34*) for hot mixtures of magmatic gases with air, this assumption of thermochemical equilibrium is a drastic simplification of the early-stage plume. Cooling and mixing of magmatic gases with air happens on similar timescales as fast chemical conversions, and by that, the assumptions of thermochemical equilibrium break down (*20*, *22*). A clear model deficiency arises when considering redox pairs: Thermochemical equilibrium models predict near-complete oxidation of reduced species such as H_2_, CO, and H_2_S in mixtures of magmatic gases with air (*23*, *36*, *37*), despite the observed presence of these reduced gases in many volcanic plumes [see, e.g., (*19*, *26*, *40*, *41*) and fig. S1]. Thus, thermochemical equilibrium models cannot provide quantitative representations of the early-stage high-temperature plume oxidative processing.

Considering chemical kinetics and parameterizing the turbulent mixing and cooling processes offers a potential solution to the dilemma of observations of BrO in plumes that contain reduced gases. Because plume cooling by dilution can be extremely fast, it is likely that the oxidation reactions of some reduced species are too slow to keep up with fast cooling, leading to a partial preservation of reduced species in the cooled plume similar to the magmatic composition at emission while still promoting sufficient halogen oxidation. Furthermore, an oxidation-production balance can be established, also leading to a persistence or even a formation of reduced species, e.g., through the interaction between carbonyl sulfide (COS) and CO [see (*20*)]. Clearly, there is a need for more comprehensive and extended modeling of the interaction of hot magmatic gases with atmospheric air that fully captures the dynamics of the coupled chemical and physical processes, in contrast to thermochemical equilibrium assumptions.

**Fig. 1.**
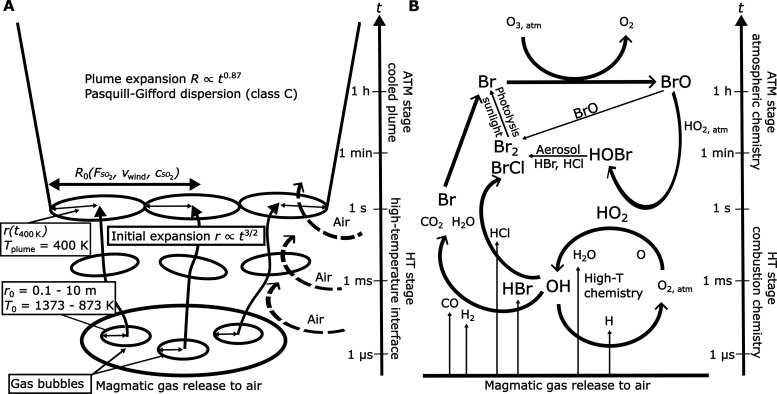
Schematic of modeled processes across the high-temperature stage (HT) and atmospheric stage (ATM) of the plume model as a function of time *t*. (**A**) Mixing scheme of the model. In the HT stage, the magmatic gas is emitted to air from a source with a radius *r*_0_ (0.1 to 10 m) (referred to as magmatic gas bubble radius) and a magmatic gas temperature *T*_0_ (823 to 1323 K), defined as the gas temperature on entering the atmosphere. Hot magmatic gases mix with air and evolve with time *t* according to chemical combustion kinetics and turbulent mixing (plume radius *r* ∝ *t*^3/2^). Once the gases cool to 400 K (*t*_400K_), the model switches to the ATM stage. Several HT stage plumes combine into an ATM stage plume with initial radius *R*_0_, derived from the SO_2_ flux $F_{SO_{2}}$, wind speed *v*_*wind*_, and the SO_2_ concentration $c_{SO_{2}}$ (from the HT stage output). The ATM plume radius *R* evolves according to the Pasquill-Gifford dispersion scheme C (*R* ∝ *t*^0.87^). The ATM stage simulates halogen multiphase photochemistry up to hours downwind. (**B**) Overview of key chemical conversions as derived from the simulations. The HT stage of the model reaction cycles involving atmospheric O_2,atm_ and hot magmatic H_2_O form OH and HO_2_. The OH reacts with HBr to form reactive bromine species (e.g., Br, BrCl, and Br_2_). These transform to BrO in low-temperature catalytic reaction cycles, destroying atmospheric O_3_. The oxidative processing of magmatic gases at the high-temperature interface is essential to generate BrO at levels observed in volcanic plumes. The (partial) oxidation of other magmatic gas species (e.g., H_2_ and CO), which have to traverse the same interface, affects the redox composition of the magmatic gases in the plume compared to the original magmatic gas emission.

Through the further development of a kinetic model of the early volcanic plume, this study seeks to more accurately quantify chemical processes at the high-temperature atmospheric interface, toward an improved linking of plume gas composition measurements to magma state and hence volcanic activity. This work builds on and expands the model approach in (*20*), which simulated C-H-O-S chemical kinetics in the hot, rapidly diluting, and cooling early-stage plume (plume age < 10 s). Our advancements include incorporating bromine, chlorine, mercury, and reactive nitrogen chemistry schemes, alongside an updated C-H-O-S scheme. We furthermore connect the hot plume mechanism based on combustion chemistry with a cooled plume mechanism based on multiphase photochemistry including the “bromine explosion” in a continually dispersing plume (plume age up to hours). This integrated model allows us to simulate the complete suite of kinetic oxidative processes of C-H-O-S-Br-Cl-N-Hg from hot gas emission to the cooled plume (see Fig. 1) and thereby quantifies the atmospheric processing of magmatic gases in relation to observations of BrO in volcanic plumes and constrains how high-temperature oxidative processes at the atmospheric interface can alter the redox composition of magmatic gas emissions.

We apply the model in a case study of Mt. Etna volcano, Italy, for which numerous observations of plume composition exist. Specifically, the long observation history at Mt. Etna provides datasets of BrO/SO_2_ at various distances downwind (*42*), in-plume O_3_ depletion (*35*, *43*), and measurements of volcanic gas redox pairs by remote sensing Fourier transform infrared spectroscopy (FTIR), e.g., CO/CO_2_ (*8*, *40*), and in situ sensors for H_2_/H_2_O and H_2_S/SO_2_ (*19*, *26*, *41*).

## RESULTS

### Physical and mixing parameters indicate a highly dynamic high-temperature atmospheric interface

Our model of volcanic plume composition simulates the chemical evolution of the plume using two stages within a single overarching model framework (see Fig. 1A and Materials and Methods for details). In the high-temperature stage (HT), hot magmatic gas is released to the atmosphere where it rapidly cools, mixes, and reacts with air. The HT stage includes combustion chemistry coupled to a fast turbulent dispersion scheme to simulate the hot magmatic gas plume as it cools down to a temperature of 400 K within seconds. Mixing is parameterized with the initial spatial scale of the gas source *r*_0_. In the following, it is referred to as magmatic gas bubble radius, linking the HT stage plume initialization to the magmatic degassing conditions. Mixing with air increases the plume radius *r* in the HT stage and is faster for emissions with smaller magmatic gas bubble radius [see also (*20*)]. We refer to this early part of the plume as the high-temperature atmospheric interface.

**Fig. 2.**
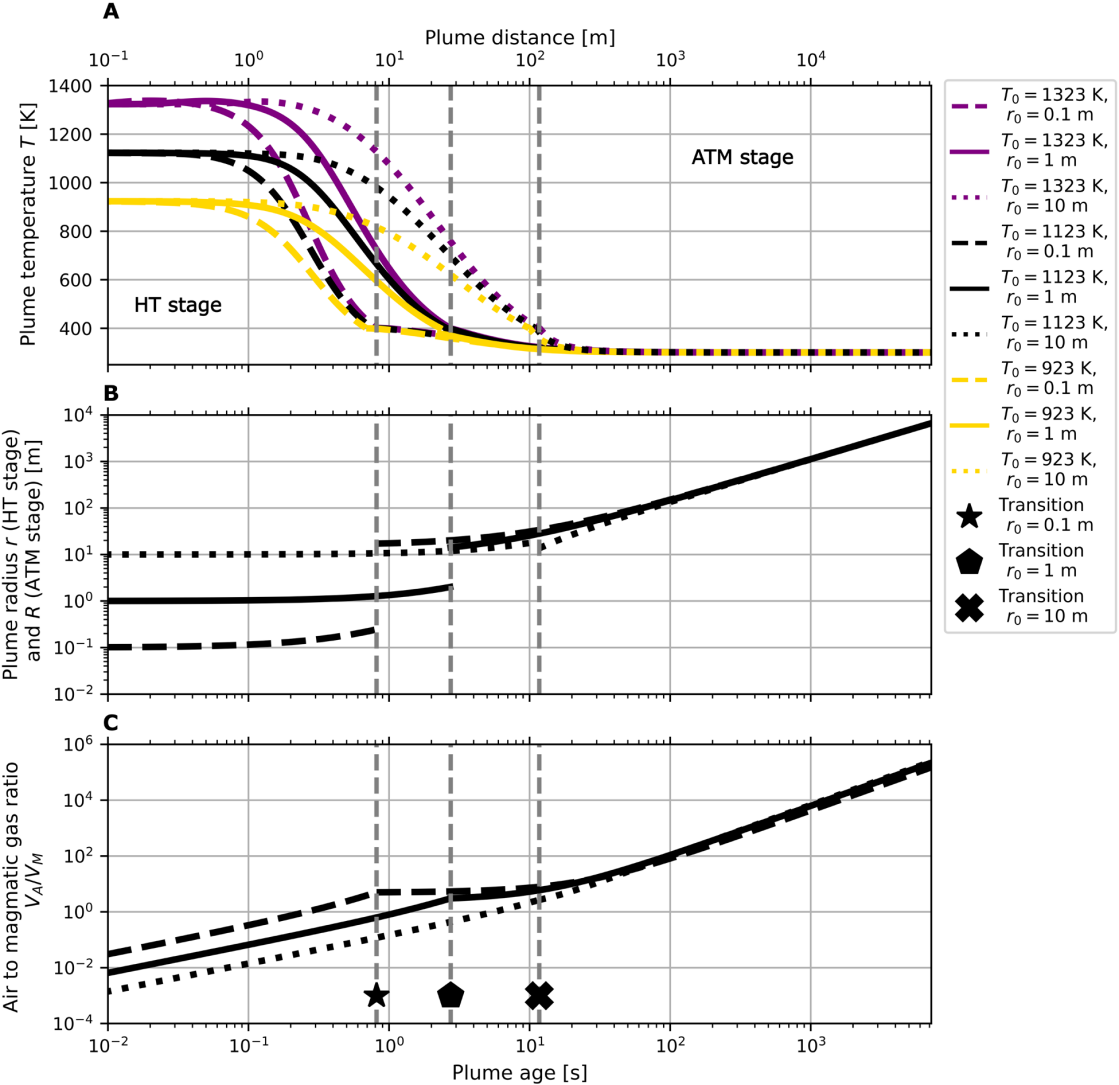
Physical parameters of the modeled turbulent mixing scheme as a function of plume evolution. Physical parameters evolution according to plume age (lower *x* axis, in [s]), which is also shown as equivalent distance for an assumed wind speed of 10 m s^−1^ (upper *x* axis, in [m]). Nine simulations are presented across three magmatic gas temperatures [*T*_0_ = 1323 K (purple lines), 1123 K (black lines), and 923 K (gold lines)] and three magmatic gas bubble radii/mixing scenarios (*r*_0_ = 0.1, 1, and 10 m). The black markers and the gray vertical lines show the 400 K transition region between the HT and the ATM stages of the model. (**A**) Plume temperature, *T*. (**B**) Plume radii [*r* (HT stage) and *R* (ATM stage)]. (**C**) Ratio of an atmospheric air volume *V*_A_ mixed into the initial volume of magmatic gas *V*_M_. Because mixing only depends on the plume radius (and not temperature), (B) and (C) show only one line for each mixing scenario.

After cooling to 400 K, multiple HT plumes combine to form a larger plume with radius *R* (scaled to represent gas emission fluxes) in the atmospheric stage (ATM). At this stage, the model simulates the subsequent chemical evolution of the cooled plume over minutes to hours downwind. It is initialized with the gas composition output from the HT stage and incorporates a multiphase photolytic halogen chemistry mechanism coupled to a slower plume dispersion parameterization. We consider 45 simulations, which reflect conditions at Mt. Etna (see the Materials and Methods) and which cover a wide parameter space for six magmatic gas temperatures (*T*_0_, defined as the gas temperature on entering the atmosphere), and HT stage and ATM stage mixing scenarios parameterized by the magmatic gas bubble radii (*r*_0_), wind speed (*v*_*wind*_), and SO_2_ gas flux ($F_{SO_{2}}$). Figure 2 shows the three physical parameters of the plume in the model represented by plume temperature *T*, plume radius *r* (HT stage), and *R* (ATM stage) and the atmospheric air–to–magmatic gas ratio (*V*_A_/*V*_M_).

These plume physical parameters exhibit large changes within the first seconds after emission. Cooling in the HT stage is driven by fast turbulent mixing of the hot magmatic gases with ambient air. All source radii (magmatic gas bubble radii) result in plume cooling down to 400 K in less than about 10 s, with cooling much faster (~x10) for the smallest magmatic gas bubble radius than the largest. The gray vertical lines in Fig. 2 denote the 400 K transition point between the HT and the ATM stages of the model. The location of this transition depends predominately on the mixing scenario and is found at plume ages of ~0.8 s (*r*_0_ = 0.1 m), 1.5 s (*r*_0_ = 1 m), and 10 s (*r*_0_ = 10 m). As mentioned above, the step increase in plume radius (visible for *r*_0_ = 0.1 and 1 m) at this transition point can be interpreted as the coalescence of multiple HT stage plumes into a single atmospheric plume in the ATM stage. The number of high-temperature plumes that are combined at the end of the HT stage depends on the model physical parameters and ranges from 5 (*T*_0_ = 923 K, *v*_*wind*_ = 15 m s^−1^, $F_{SO_{2}}$ = 15 kg s^−1^) to 28 (*T*_0_ = 1323 K, *v*_*wind*_ = 5 m s^−1^, $F_{SO_{2}}$ = 5 kg s^−1^) for the *r*_0_ = 1 m HT stage runs. Air entrainment, as traced by increasing *V*_A_/*V*_M_, is extremely fast in the HT model stage compared to the ATM stage (order-of-magnitude changes in *V*_A_/*V*_M_ within seconds in the former but minutes to hours in the latter). This is consistent with the respective *t*^3/2^ and *t*^0.87^ time dependencies of the plume radii (*r* and *R*) according to the turbulent mixing parameterizations in these model stages. The rapid decrease in temperature and increase in *V*_A_/*V*_M_ occurring within seconds reflects the highly dynamic nature of the near-source plume processes (HT model stage), which cannot be resolved by previous approaches based on thermochemical equilibrium calculations at fixed *V*_A_/*V*_M_ and temperature [e.g., (*36*, *37*)]. Critical to the plume chemical evolution is the coupled temperature and *V*_A_/*V*_M_ pathway as the in-mixing of oxidant-rich air via increasing *V*_A_/*V*_M_ promotes oxidation reactions but the concurrent decrease in temperature slows down most chemical reactions. These competing factors exert important controls on the plume chemistry during the HT stage, in particular via the formation of reactive HO*_x_* (OH and HO_2_) radicals, which act as key plume oxidants (see below). In the following, we investigate plume chemistry of simulations with an initial emission radius of *r*_0_ = 1 m (solid lines in Fig. 2) and focus on three representative magmatic gas temperatures (*T*_0_ = 1323, 1123, and 923 K). Results and processes for the other mixing scenarios (e.g., different magmatic gas bubble radii, wind speeds, and SO_2_ fluxes, tested across 45 simulations) are fundamentally similar (see figs. S2 to S6 and S8).

### High-temperature atmospheric oxidation can substantially modify the emitted magmatic gas composition

**Fig. 3.**
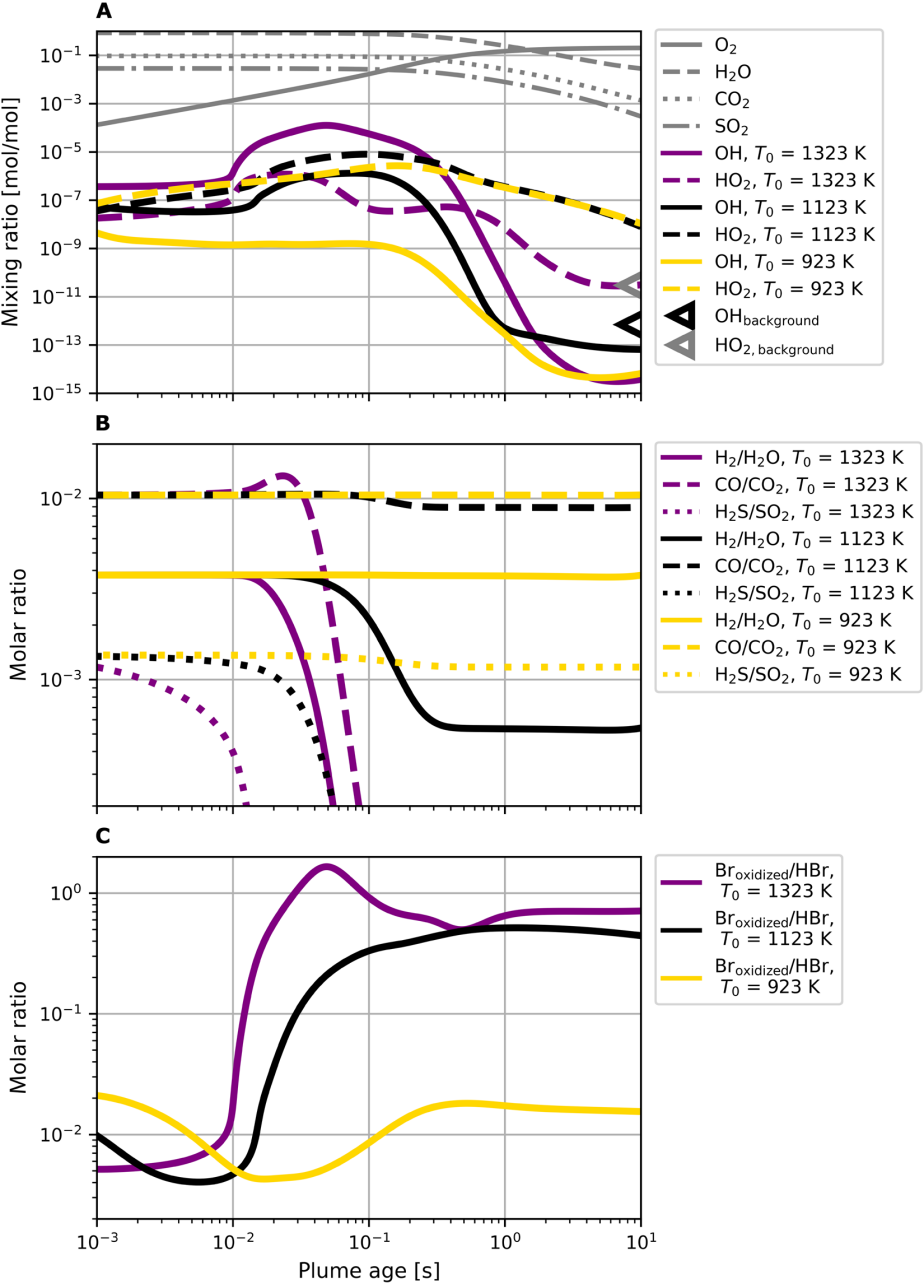
Chemical evolution of gas composition during mixing with air in the first 10 s after magmatic gas emission. Results for the HT model stage, for three different magmatic gas temperatures [*T*_0_ = 1323 K (purple lines), *T*_0_ = 1123 K (black lines), and *T*_0_ = 923 K (gold lines)] and for a magmatic gas bubble radius of *r*_0_ = 1 m. (**A**) Evolution of the main emitted species (H_2_O, CO_2_, and SO_2_; gray lines), in-mixed O_2_ (gray solid line), and corresponding production of main HO*_x_* radicals (OH and HO_2_). The markers on the right edge of the plot indicate the atmospheric background mixing ratios of OH_background_ and HO_2,background_. (**B**) Main redox couples in the HT stage (H_2_/H_2_O, CO/CO_2_, and H_2_S/SO_2_) are shown with the same color coding as in (A). (**C**) Ratio between oxidized bromine species (Br_oxidized_) and the reduced bromine species HBr, which is the main bromine gas species in the emission prior to mixing with the atmosphere.

The potential for rapid oxidation of magmatic gases in volcanic plumes is driven by the formation of HO*_x_* (OH and HO_2_), a key atmospheric oxidant. High-temperature generation of HO*_x_* occurs within seconds after magmatic gases enter air (Fig. 3). At high plume temperatures (>1000 K), the in-mixing of atmospheric O_2_ triggers an exponential production cycle of HO*_x_* radicals in the plume. It is driven by the reaction of atmospheric O_2_ with hot water vapor from the volcanic emission and strongly depends on temperature (*20*, *22*). This increase in HO*_x_* contrasts to the temporal evolution of the mixing ratios of the major volcanic gas species (H_2_O, CO_2_, and SO_2_), which are, due to their abundance, mainly controlled by dilution (gray lines in Fig. 3A).

The formation of HO*_x_* radicals in the three HT stage plume simulation runs (*T*_0_ = 1323, 1123, and 923 K with magmatic gas bubble radius *r*_0_ = 1 m) shows a distinct pattern with magmatic gas temperature. At high temperatures, there is a preferential production of OH [tens of parts per million (ppm) for the emission temperature of 1323 K], which is converted into the more stable HO_2_ in later plume stages (*t* > 0.1 s). For lower magmatic gas temperature scenarios, maximum OH reaches parts per billion (ppb) levels, and HO_2_ production dominates over OH (see black and gold dashed lines in Fig. 3A). All model runs find that net OH production is transient, lasting some tenths of a second. For the lower magmatic gas temperatures, HO_2_ persists in the plume at amounts that exceed atmospheric background of 20 parts per trillion (ppt) by orders of magnitude.

The formation of HO*_x_* oxidants in the HT stage affects the evolution of reduced magmatic gas species in the early plume (Fig. 3, B and C) as the OH radical is highly reactive to reduced magmatic gases. During the short period of cooling from magmatic to atmospheric temperatures, reduced species can be strongly oxidized, leading to the depletion of H_2_ and H_2_S in the plume for the high-temperature emission scenarios and, to a large extent, remove CO, following its transient net formation. At lower magmatic gas temperatures (*T*_0_ < 1100 K), and thus lower OH radical amounts, the reduced magmatic gas species are oxidized much less and thus better preserve the emitted ratios of redox couples.

The behavior of C-H-O-S species simulated with our extended high-temperature chemical mechanism show excellent consistency with the results in (*20*), which used a previous version of the HT stage of our model and only considered C-H-O-S chemistry.

Figure 3C shows the impact of the HT stage HO*_x_* on the evolution of volcanic bromine. HBr, just as H_2_, CO, and H_2_S, is a reduced magmatic species, which is oxidized by reaction with OH. We next demonstrate how high-temperature oxidation of HBr in the HT stage plume influences the formation of BrO in the cooled plume, as frequently measured in many volcanic plumes including Mt. Etna.

### High-temperature atmospheric oxidation is essential for the rapid formation of BrO in the plume

**Fig. 4.**
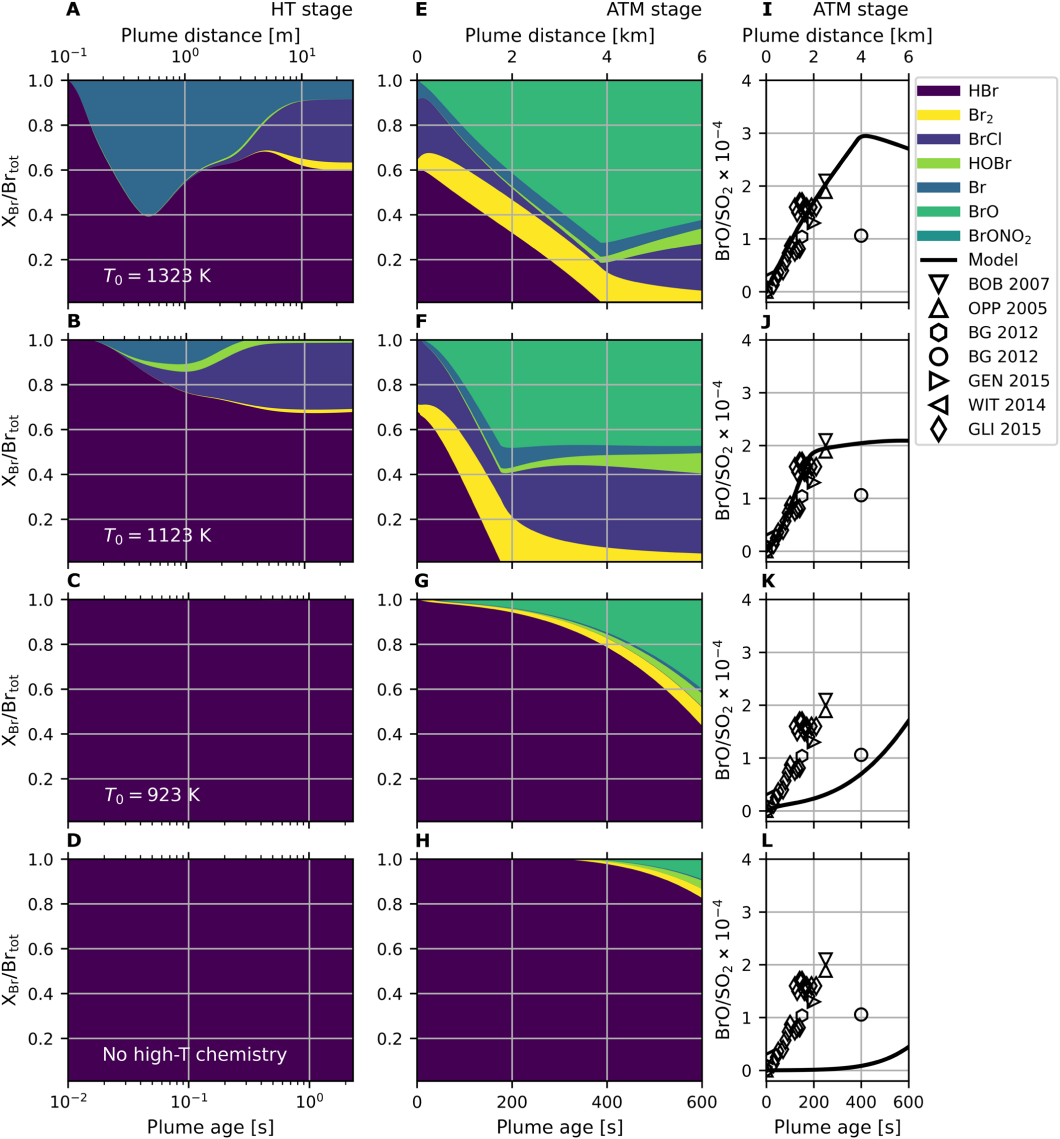
Overview of relative bromine speciation and BrO/SO_2_ ratios simulated across HT and ATM stages. The evolution of relative bromine speciation (X_Br_/Br_tot_) and BrO/SO_2_ of the model as a function of time (lower *x* axis, [s]) and equivalent distance (upper *x* axis, [m] or [km]). The different rows correspond to different magmatic gas temperatures (row A: *T*_0_ = 1323 K, row B: *T*_0_ = 1123 K, row C: *T*_0_ = 923 K, and row D: reference run without high-temperature chemistry in the HT stage). The first column (**A** to **D**) shows the evolution in relative bromine speciation during the HT plume stage through cooling to 400 K. Note the logarithmic time axis. The second column (**E** to **H**) shows the relative bromine speciation during the ATM stage, which has been initialized with output from the HT stage, during the first 10 min of plume evolution (with linear time axis). The last column (**I** to **L**) shows the BrO/SO_2_ ratio for these simulation runs (black lines) compared to near-source (distance < 6 km, equivalent to <600 s for a wind speed of 10 m s^−1^) plume observations (open markers) from the compilation in (*42*). Results for the intermediate temperatures *T*_0_ = 1223, 1023, and 823 K and for the mixing scenarios with magmatic gas bubble radius *r*_0_ = 0.1 and 10 m are shown in figs. S4 to S6.

The HO*_x_* radicals, formed during the high-temperature mixing process react rapidly with magmatic hydrogen halides (HBr and HCl) in the early plume. Considerable amounts of oxidized halogen species (referred to as reactive halogens) are formed within less than 0.1 s. Further speciation changes and bromine oxidation occur as the plume cools. The chemistry in the cooled plume quickly produces high amounts of BrO (within minutes; see Fig. 4, I to K where BrO is shown relative to SO_2_ that acts as a plume tracer). The modeled BrO/SO_2_ ratios for high emission temperatures (e.g., 1323 and 1123 K) reach up to 3 × 10^−4^ within minutes in the downwind plume (distance < 6 km), comparable to numerous remote sensing observations that persistently measure BrO at varying activity at Mt. Etna [data from the compilation in (*42*) and references therein]. The accuracy of our model is further confirmed by the modeled ozone loss in the plume, which agrees with measurements reported in the literature (see fig. S7). Moreover, we can demonstrate that the fast formation of BrO (rise in BrO/SO_2_ over the first 6 km) in our model is largely independent of atmospheric dynamics (see fig. S8). In contrast, the reference run, which ignores high-temperature chemistry, substantially underestimates observed BrO (Fig. 4 K). Also, at 923 K, the emission temperature is too low to form the required amounts of reactive halogens and, consequently, the formation of BrO is much slower than observed.

Figure 4 (A to C) shows an overview over the bromine speciation during the HT stage. The most relevant chemical reactions involved in the formation of BrO in volcanic plumes are summarized in Fig. 1B and tables S1 (HT stage) and S2 (ATM stage) in the Supplementary Materials. At high temperatures, the reaction of halogen halides with OH produces Br and Cl radicals. Further speciation changes occur within seconds, e.g., formation of hypobromous acid (HOBr), bromine chloride (BrCl), and dibromine (Br_2_) depending on the initial gas composition, temperature, and mixing. BrO amounts remain low during the HT stage, and for the Mt. Etna case, the major reactive bromine species that persists after the plume cools is BrCl (see Fig. 4, A to C), formed predominately by the reaction of Br with Cl_2_. This is due to the high reactivity of bromine and the abundance of chlorine. The HCl/HBr ratio in the magmatic gas emission is ~1000, whereas the chlorine reactivity is lower by a similar factor, yielding comparable amounts of reactive chlorine and bromine (see fig. S9). Beyond that, chlorine is much less reactive than bromine in the cooled plume; therefore, chlorine chemistry is not further discussed in detail. The produced reactive bromine species persist to the end of the HT stage, as the plume evolves, dilutes, and cools to 400 K. At this point, ~40% of the emitted HBr has been converted into reactive bromine for the 1323 K emission scenario and ~30% for the 1123 K emission temperature.

The fraction of reactive bromine further increases in the ATM simulation stage through autocatalytic atmospheric chemistry cycles involving acidic aerosol and sunlight [also known as “bromine explosion” (*31*)]. It can reach up to 100% within a few minutes in the high-temperature emission scenarios (see Fig. 4, E and F). Soon, the most abundant species is BrO followed by BrCl, Br_2_, and HOBr. The initially formed reactive halogen species kick-start this autocatalytic formation of BrO and the associated destruction of O_3_. BrCl and Br_2_ are photolyzed and enhance the availability of Br and Cl radicals, which rapidly react with atmospheric O_3_ to form BrO and ClO, respectively. To recycle Br atoms, BrO either self-reacts if concentrations are sufficiently high or forms HOBr upon reaction with HO_2_. HOBr partitions to the liquid particle phase and reacts with $Br^{-}$ (or $Cl^{-}$) ions, originating from the ionic decomposition of halogen halides in aerosol particles. The reaction produces volatile Br_2_ (or BrCl), which partitions back to the gas phase and, after photolysis, provides two halogen radicals, which again form BrO. This autocatalytic formation of reactive bromine can explain the further oxidation of HBr at atmospheric temperatures [see, e.g., (*44*)] that is extremely fast in volcanic plumes. The high-temperature formation of HO_2_ and the persistence of enhanced HO_2_ levels as the plume cools (Fig. 3A) initially drive the formation of HOBr and subsequently BrO (see Fig. 1B) in the early plume, with entrainment of background HO_2_ sustaining HOBr-BrO chemistry as the plume dilutes.

We summarize that HO*_x_* radicals produced in the high-temperature atmospheric interface are essential to explain the observed fast formation of BrO in volcanic plumes. High HO*_x_* levels both lead to the initial oxidation of HBr and provide the HO_2_ to kick-start heterogeneous chemistry resulting in early formation and high BrO amounts in the cooled plume.

### Impact of the high-temperature atmospheric interface on redox composition of the plume

Our model results indicate a critical role of high-temperature HO*_x_* formation in the oxidation of magmatic bromine emissions. These HT stage oxidation processes occur over very short scales (seconds or less, i.e., centimeters to meters) at the atmospheric interface. The model provides a comprehensive and quantitative explanation of the coupled chemistry and mixing processes that convert magmatic HBr to the observed BrO levels in volcanic plumes. On the basis of these findings, we can now investigate the fate of other reduced magmatic gas species, which have to pass through this same high-temperature atmospheric interface.

**Fig. 5.**
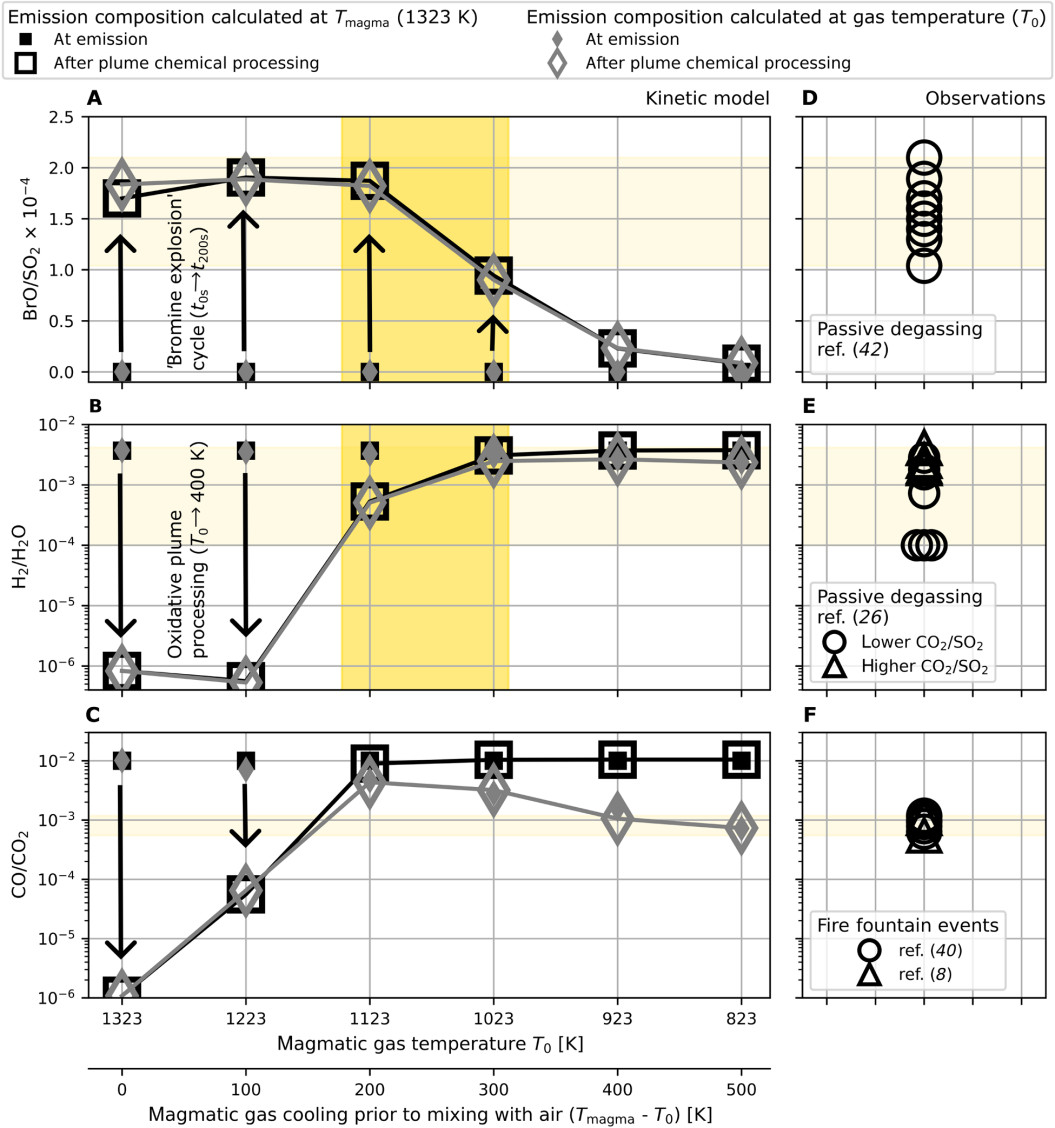
Simulated BrO/SO_2_ at 2 km downwind and redox gas ratios in the cooled plume compared to plume observations. Simulations of BrO/SO_2_ at 2 km downwind and redox couples in the cooled plume (plume temperature below 400 K) are performed across a range of magmatic gas temperatures *T*_0_ corresponding to varying amounts of magmatic gas cooling prior to mixing with air. The left column (**A** to **C**) shows the model results after plume chemistry (open markers) compared to the composition at emission (closed markers) as used in the model initialization. Shown are two simulation variants: Black markers correspond to simulations with a constant gas composition released at the magmatic gas temperature *T*_0_, and gray markers show simulation runs where the emission composition of H_2_/H_2_O and CO/CO_2_ are buffered by the nickel-nickel-oxide (NNO+0.35) magmatic redox buffer. The right column (**D** to **F**) shows field observations of Mt. Etna plume composition under varying volcanic activity (and for H_2_/H_2_O is denoted by a comeasured CO_2_/SO_2_ molar ratio above or below 1.2). The faint shaded area marks correspondence between model results and plume observations, whereas the darker shade marks the temperature range where BrO formation and H_2_/H_2_O together correspond to observations in the passive degassing Mt. Etna plume.

Our results on halogen processing along with BrO observations in volcanic plumes provide a constraint to the high-temperature atmospheric interface, specifically a minimum magmatic gas temperature threshold needed to produce the observed rapid formation of BrO in volcanic plumes. In Fig. 3, we showed that higher magmatic gas temperatures can lead to the oxidation of reduced gases such as CO, H_2_, and H_2_S at the atmospheric interface. We next assess the implications of the high-temperature oxidative environment for two important redox couples in volcanic gas geochemistry, H_2_/H_2_O and CO/CO_2_ (*45*). We focus on the fate of H_2_ and CO, whose chemical reaction cycles are well constrained in contrast to those of sulfur (see fig. S10) (*22*, *46*–*49*). Figure 5 (A to C) shows the plume composition after chemical processing for model initializations at six magmatic gas temperatures (*T*_0_ from 1323 to 823 K), assuming either (i) unreactive magmatic gas cooling prior to its release to air or (ii) a closed system where the magmatic gas composition in H_2_/H_2_O and CO/CO_2_ is controlled by the nickel-nickel-oxide (NNO+0.35) redox buffer at each magmatic gas temperature. We compare the model results to plume measurements of BrO/SO_2_ ratios (*42*) (Fig. 5D) and to the few available reported field measurements of H_2_/H_2_O (*26*) and CO/CO_2_ (*8*, *40*) redox gas couples in the plume of Mt. Etna at varying volcanic activity (Fig. 5, E and F).

Volcanic plumes containing BrO, such as Mt. Etna, must arise from a sufficiently hot and oxidizing atmospheric interface, with magmatic gas emission temperature *T*_0_ > ~1000 K (Fig. 5A). Cooler gas emissions (*T*_0_ < ~1000 K) retain the redox imprint of the pure magmatic gas but fail to reproduce the plume BrO observations. The high-temperature oxidation chemistry that is necessary to form BrO can substantially modify the plume redox composition. Redox couple gas ratios are most strongly affected at higher magmatic gas temperatures with H_2_/H_2_O affected more readily than CO/CO_2_. This order reflects relative net reactivity of these reduced gases with OH, which is most abundant in hotter plumes (Fig. 3).

Measurements of H_2_ in the plume of Mt. Etna have been taken from the crater rim with an electrochemical in situ sampler and exhibit a high variability, with H_2_/H_2_O ranging from 10^−4^ to 10^−2^ (*26*). This has been interpreted as reflecting two distinct emission sources, with the H_2_-rich component arising from a magmatic reservoir at 1323 K controlled by an NNO+0.35 redox buffer. The H_2_-poor component cannot be explained using an NNO+0.35 redox state; instead, (*26*) proposes a more oxidized (NNO+2.3) conduit gas composition that is released to the atmosphere at 823 K. On the basis of our model results, we expect that plume chemistry would substantially affect the 1323 K gas emissions, with H_2_ largely depleted by oxidation at the high-temperature interface (Fig. 5). Thus, the H_2_-rich plume is unlikely to reflect emissions released at 1323 K to the atmosphere. The emission would require cooling to less than ~1123 K prior to mixing with air for the magmatic H_2_/H_2_O redox signature to be preserved. Yet, the observed BrO formation can only occur if the magmatic gases were hotter than 1023 K at the high-temperature atmospheric interface when they first came into contact with air. We identify a region of magmatic gas temperatures of *T*_0_ = 1023 to 1123 K that meet these criteria, where the model reproduces the observed BrO/SO_2_ and H_2_/H_2_O in the passively degassing Mt. Etna plume (Fig. 5). The H_2_-rich gas component is reported to be associated with greater CO_2_ abundance, indicative of a deeper gas source (Fig. 5E) (*26*). In this context, a plausible mechanism for gas cooling prior to emission could be the adiabatic cooling and expansion of CO_2_-rich and H_2_-rich rising magmatic gas bubbles or cooling of the gas as it rises in the conduit [see, e.g., (*13*, *14*, *50*)]. These processes could theoretically lead to several hundred kelvin of cooling (*13*) prior to mixing of the magmatic gases with air. Recently, (*14*) applied gas buffer conditions to the dataset in (*26*) to infer amounts of magmatic gas cooling of 180 to 306 K (H_2_-rich component) and 555 to 558 K (H_2_-poor component) at Mt. Etna. This magnitude of cooling of the H_2_-rich component is consistent with the *T*_0_ = 1023 to 1123 K region identified by our model, which corresponds to 200 to 300 K below magmatic temperature. Our model thereby provides independent constraints to quantify emission temperatures (of magmatic gases entering air) and the degree of decoupling between magmatic gas bubbles and magmatic temperature. Moreover, our model results suggest that the H_2_-poor (and CO_2_-poor) component could alternatively actually reflect strong oxidation at a high magmatic gas temperature at the atmospheric interface.

Measurements of CO in the Mt. Etna plume are reported for fire fountain activity, where FTIR measurements yield CO/CO_2_ ~ 10^−3^ (*8*, *40*). This ratio has been interpreted as the emitted gas composition from an ~NNO Etna magma cooled to 803 to 913 K (*8*) and is consistent with our model finding that plume chemistry processes negligibly affect CO/CO_2_ at such low magmatic gas temperatures. The fire fountain was reported in (*8*) to be several tens of meters wide and consisted of a “red-orange core of incandescent gas and molten lava clots (~1373 K), surrounded by a darker envelope of finer pyroclasts (cinders and lapilli) and gas.” The FTIR sampled the outer region, in which the temperature of the fine pyroclasts was measured to be 783 to 903 K. Our model indicates that the CO/CO_2_ redox composition of the hotter inner core would be strongly affected by plume chemistry, becoming CO depleted by oxidation. According to our model results, it is plausible that the measured gas composition of the outer region reflects gases emitted somewhat above 1123 K, where emitted CO levels could have been initially higher but then considerably lowered through oxidation processes at the atmospheric interface [see also (*20*)], during cooling and dilution of the fountain edge.

## DISCUSSION

### The high-temperature atmospheric interface relates the plume composition to the redox state of the emitted magmatic gases

On emission and mixing with atmospheric air, hot magmatic gases undergo rapid chemistry. Oxidative processing of reduced magmatic gases (HBr, as well as H_2_ and CO) at the high-temperature atmospheric interface leads to the formation of BrO in the plume and can modify the magmatic gas redox composition. Our kinetic model quantifies these processes, finding that the magmatic gas temperature exerts critical control on the extent of oxidation, which is reflected in the resulting plume composition (BrO and ratios of redox pairs).

OH radicals, formed as atmospheric O_2_ intrudes the hot H_2_O-rich magmatic gas mixture, play a prominent role in oxidizing HBr emissions and ultimately provide enhanced levels of HO_2_ in the cooled plume. These support the rapid heterogeneous autocatalytic production of BrO in the downwind plume. The production of OH and HO_2_ radicals at the atmospheric interface is orders of magnitude slower at lower emission temperatures, preventing major formation of BrO. Thus, plume BrO observations can demark a lower limit for the temperature of volcanic gas emissions to the atmosphere, which is *T*_0_ > ~1000 K for this Mt. Etna case study.

Alongside HBr, other reduced magmatic gases such as H_2_ and CO must also pass through the same oxidizing high-temperature interface. Our study shows that also their fate depends critically on the amount of OH radicals at the atmospheric interface and thus the temperature of the gas at emission to air. The modeled oxidation of reduced gases at high magmatic gas temperatures above ~1100 K is consistent with specific field observations, e.g., of H_2_ burning at Kilauea (*24*). Nevertheless, the many observations of these reduced gases in cooled plumes [e.g., (*8*, *26*, *40*)] provide evidence that these species at least partially survived oxidation at the high-temperature atmospheric interface. Confronting our model with specific observations of H_2_/H_2_O hence demarks an upper limit to the magmatic gas temperature at emission to the atmosphere, which is *T*_0_ < ~1100 K for this Mt. Etna case study.

Using these upper and lower limits, we can identify magmatic gas temperature conditions that allow observed BrO amounts to form in the volcanic plume and for which H_2_ and CO also survive chemical processing at the high-temperature atmospheric interface. At Mt. Etna, cooling by 200 to 300 K below magmatic temperature (1323 K) prior to emission or contact to the atmosphere would slow down high-temperature oxidation of the reduced gas species to preserve the concentrations of H_2_ and CO at the levels observed in the Etna plume yet still enable sufficient BrO formation. Our plume chemistry model results thereby provide evidence supporting hypothesized decoupling of gas temperatures from magma due to cooling associated with adiabatic gas bubble expansion in the melt or gas cooling in the conduit (*13*, *14*).

Our study focuses on the plume of Mt. Etna (chosen as the best observed), but investigations can be widened to other plume case studies such as Masaya (Nicaragua), which temporarily hosted a lava lake and thus had a direct high-temperature atmospheric interface accessible for measurements of reduced species and reactive halogens (*25*, *51*). Although BrO observations are persistent at Mt. Etna, indicative of hot gas emissions, our model finds that cooler gas emissions below ~1000 K cannot produce considerable BrO. This finding is consistent with field observations at Cotopaxi (Ecuador) that trace the appearance of detectable BrO in the plume as the initially hydrothermal source became more magmatic (*52*). This transition could have led to increased gas emission temperatures at the atmospheric interface with sufficient high-temperature oxidative halogen processing. We note that increasing temperatures may also diminish halogen scrubbing in the Cotopaxi hydrothermal system. Nevertheless, the presence of HCl (and by inference likely also HBr) is reported before the appearance of BrO (*52*), consistent with the notion that further temperature increases could have led to the development of an oxidative high-temperature atmospheric interface and thus the formation of BrO.

Recent works (*14*, *15*) have emphasized the strong potential for observations of redox gas couples in volcanic plumes to inform on magmatic conditions and volcanic activity. Our model framework can enable such datasets to be probed more deeply through the consideration of high-temperature atmospheric processes on redox gas composition and in forming plume BrO. The model also provides a wider range of interpretations of reported redox gas measurements than can be determined from gas-phase thermochemical equilibria and magmatic gas-melt-rock interactions alone. For instance, we can determine under which dynamic conditions rising magmatic gas bubbles sustain thermochemical equilibrium within the melt, as widely assumed. In the future, we can adapt our model framework to additionally trace the chemical kinetics in the magmatic gas bubble prior to their release to air. Moreover, the model allows the study of scenarios where the observed redox composition may arise from gases emitted at higher temperatures before undergoing a degree of oxidative processing [see also (*20*)]. Small variations in the magmatic gas temperature at emission to air can thus drastically alter the redox ratios in the cooled plume. The magmatic gas temperature likely varies as a function of the pathway of magmatic gas bubbles in the subsurface and the form of the high-temperature interface (e.g., direct contact of magma with air at a lava lake or fire fountain event, or via magmatic gas bubble rising with adiabatic expansion and cooling prior to entering air, within magma or within the gas-filled conduit). We suggest that combined measurements of reduced magmatic species and oxidized halogens have the potential to inform about the state of the high-temperature atmospheric interface as a function of volcanic activity changes. The discussion of the fate of H_2_S and other sulfur compounds in this study was limited by uncertainties in the high-temperature reactions of sulfur that are the focus of current laboratory experiments [e.g., (*47*–*49*, *53*)]. Incorporating these findings into our model in the future will provide vital additional information.

In general, more high-quality (low-interference, accurately corrected for atmospheric influence) measurements of reduced magmatic gases in volcanic plumes are needed to constrain our model. Ideally, they are measured alongside BrO, which is already monitored at some volcanoes by ground-based stations (*54*, *55*) and by satellite globally (*56*).

By tracing the oxidation processes that form BrO in volcanic plumes, our kinetic model can support the interpretation of continuous BrO data as recorded at some volcanoes. An improved understanding of the influences of meteorological conditions and volcanic activity on plume BrO levels (*29*) is needed to integrate plume BrO measurements into volcano hazard monitoring. Moreover, model estimates of bromine speciation in the cooled plume can be used to initialize larger-scale atmospheric chemistry and transport models to assess impacts of volcanic halogen emissions, e.g., on ozone depletion at regional to global scales (*33*, *35*).

Our model provides a quantitative link between volcanic plume and magmatic gas emission. Efforts to link observations of the composition of volcanic plumes to magmatic conditions must consider the combined effects of all relevant processes between the source and measurement, including rapid oxidizing chemistry that can occur when the hot magmatic gases come in contact with air in the very early plume, i.e., the high-temperature atmospheric interface. For volcanic plumes containing BrO near the source, the high-temperature atmospheric interface has the potential to substantially oxidize reduced magmatic gases and thus modify the redox composition of the plume.

## MATERIALS AND METHODS

### Kinetic chemistry and transport model of volcanic gas composition from hot emission to cooled plume

Our kinetic chemistry and transport model consist of two stages as indicated in Fig. 1. The HT model stage uses a C-H-O-S combustion mechanism and added submechanisms for reactive nitrogen chemistry (NO*_x_*), halogen chemistry (Cl and Br), and mercury chemistry (*57*–*60*). The ATM model stage includes a multiphase photochemical atmospheric chemistry mechanism (*17*, *34*, *35*) focusing on reactive halogens and including a simplified treatment of background atmospheric chemistry (reactive oxygen and nitrogen species). Heterogeneous gas-aerosol chemistry in the ATM stage is based on a parameterization with uptake coefficients following (*61*), and the photochemical reaction mechanism uses photolysis frequencies from the Quick TUV (Tropospheric Ultraviolet and Visible radiation) model (*62*). The high-temperature combustion mechanism is used in the HT model stage from the time of gas emission to the atmosphere until the plume cools to a temperature of 400 K (*t*_400K_). From that point, the atmospheric chemistry mechanism in the ATM stage is used and initialized with the output of the HT stage.

The turbulent mixing of the magmatic gases with atmospheric air is parameterized differently in the two model stages. In both stages, the growth of the plume radius with time *t* (or equivalently distance from the source for a given advective plume rise/propagation and wind speed) determines the rate of entrainment of atmospheric air into the plume. The HT stage of the model is characterized by fast and increasing turbulent mixing with a plume radius *r* proportional to *t*^3/2^ (*20*). Together with the generalized assumptions on atmospheric turbulence (represented by the exponent 3/2), the magmatic gas bubble radius *r*_0_ is the only parameter of the early mixing process in the model [see (*20*) for details]. A small magmatic gas bubble radius—or source radius—of the emitted gas volume (*r*_0_) also causes fast dilution of magmatic gas concentrations in air, leading to increased rates of cooling (vice versa, larger *r*_0_ leads to slower mixing, i.e., slower cooling). Thereby, applying only a few magmatic gas bubble radii can cover an extensive range of different emission conditions, small and large sources, and low and high turbulence scenarios. To some extent, they also inform about spatial inhomogeneities of the plume (plume boundary mixes faster than plume core), which are not directly resolved by our model. In the Supplementary Materials (fig. S3), we show that the magmatic bubble radius has no substantial influence on our results.

At the transition to the ATM stage, the model is reinitialized. Several HT stage plumes coalesce, resulting in the initial ATM stage plume radius *R*_0_ = ($F_{SO_{2}}$/π$c_{SO_{2}}$*v*_*wind*_)^0.5^ with $c_{SO_{2}}$ the SO_2_ mixing ratio provided by the HT stage model output, the SO_2_ flux $F_{SO_{2}}$, and the wind speed *v*_*wind*_. The plume radius, *R*, now increases more slowly, corresponding to standard atmospheric plume dispersion schemes (see below). Overall, the model is computationally fast (~1 s per stage and per model run on a personal computer), allowing for extensive sensitivity studies, and it can readily be adapted to specific volcanic systems.

### Case study of Mt. Etna emission plume

To simulate the Mt. Etna plume, the model is initialized using a high-temperature magmatic gas composition reported in (*63*), based on modeling and field observations in (*32*) and (*64*). It includes the following gas species and molar mixing ratios: H_2_O: 0.86, CO_2_: 0.096, SO_2_: 0.029, HCl: 0.014, and HBr: 1.3 × 10^−5^. The abundance of reduced gases is calculated according to a nickel-nickel-oxide (NNO+0.35) redox buffer [log(*f*O_2_) = −9.26; (*65*, *66*)] at a magmatic temperature of 1323 K (*26*), leading to the following molar ratios of redox couples: H_2_/H_2_O: 0.0037, CO/CO_2_: 0.010, and H_2_S/SO_2_: 0.0014. Trace amounts of radicals exist in the initial plume gas mixture (*36*), which are calculated according to thermochemical equilibrium at magmatic temperature, leading to the following mixing ratios of the most abundant radicals: OH: 4.7 × 10^−7^, Cl: 3.7 × 10^−7^, H: 1.2 × 10^−7^, and Br: 8.6 × 10^−8^. This emission composition forms the basis for the following sensitivity studies on plume chemistry as a function of temperature, cooling, and dilution rates: We consider six magmatic gas temperatures for the emitted magmatic gases (*T*_0_ = 1323, 1223, 1123, 1023, 923, and 823 K), and apply three emission source radii (magmatic gas emission bubble radii, *r*_0_ = 0.1, 1, and 10 m) to cover order-of-magnitude differences in the rate of plume mixing with air within the HT stage of the model. The range of initial temperatures covers an unreactive (or quenched) cooling of up to 500 K of the magmatic gases prior to their mixing with air. Additional model runs investigate the plume chemistry of a buffered gas composition emitted from a cooled magma with lower H_2_/H_2_O and CO/CO_2_ ratios according to NNO+0.35 redox buffer calculations (i.e., a closed system) across the six *T*_0_ temperatures, whereas the H_2_S/SO_2_ ratio is held constant as assumed less responsive to magma redox changes following (*11*). Also investigated is a control run with the HT stage chemistry turned off to highlight the relevance of kinetic high-temperature chemical processing.

The background atmosphere mixed into the plume has a composition representative for the Mediterranean midday summer atmosphere (including 80 ppb O_3_, 0.7 ppt OH, and 20 ppt HO_2_ as key oxidants alongside NO*_x_* and CH_4_ chemistry), which is assumed static over the simulation time of 1 hour. Atmospheric pressure and density are set according to a plume height of 3.3 km and the atmospheric temperature of 300 K. The photolysis frequencies are calculated for Mt. Etna location and elevation at midday, assuming a constant solar zenith angle for the short 1-hour simulation time. Heterogeneous reactions are only considered in the ATM stage (i.e., only in the cooled and condensed plume). We assume an aerosol surface area of 2 × 10^−11^ μm^2^ per molecule of SO_2_ (*67*). The plume dispersion parameterization in the ATM stage uses a wind speed of *v*_*wind*_ = 10 m s^−1^ and an SO_2_ flux of $F_{SO_{2}}$ = 36 kg s^−1^, which are typical meteorological and degassing conditions at Mt. Etna. We choose to apply Pasquill-Gifford dispersion class C, which gives an intermediate situation with slightly unstable conditions and an ATM plume radius (*R*) evolution proportional to *t*^0.87^ [strong solar insulation and surface wind speeds > 6 m s^−1^ (*68*)]. Figure S8 provides sensitivity of the results on $F_{SO_{2}}$ at 12 and 72 kg s^−1^ and *v*_*wind*_ at 5 and 15 m s^−1^, thereby covering a wide dispersion parameter space.

## Supplementary Materials

### This PDF file includes:

Figs. S1 to S10 Tables S1 and S2 References
