# Supplementary material for: Reactive bromine in volcanic plumes confines the emission temperature and oxidation of magmatic gases at the atmospheric interface
Source: Sci Adv. 2025 Apr 30;11(18):eadt8607. doi: 10.1126/sciadv.adt8607 (PMC12042871; doi:10.1126/sciadv.adt8607)
Supplement: Supplementary file 1 — Figs. S1 to S10 Tables S1 and S2 References [file sciadv.adt8607_sm.pdf]

Science Advances
AAAS

Supplementary Materials for

Reactive bromine in volcanic plumes confines the emission temperature and oxidation of magmatic gases at the atmospheric interface

Alexander Nies *et al.*

Corresponding author: Alexander Nies, alexander.nies@cnrs-orleans.fr;
Tjarda J. Roberts, tjarda.roberts@lmd.ipsl.fr

Sci. Adv. **11**, eadt8607 (2025)
DOI: 10.1126/sciadv.adt8607

This PDF file includes:

Figs. S1 to S10
Tables S1 and S2
References

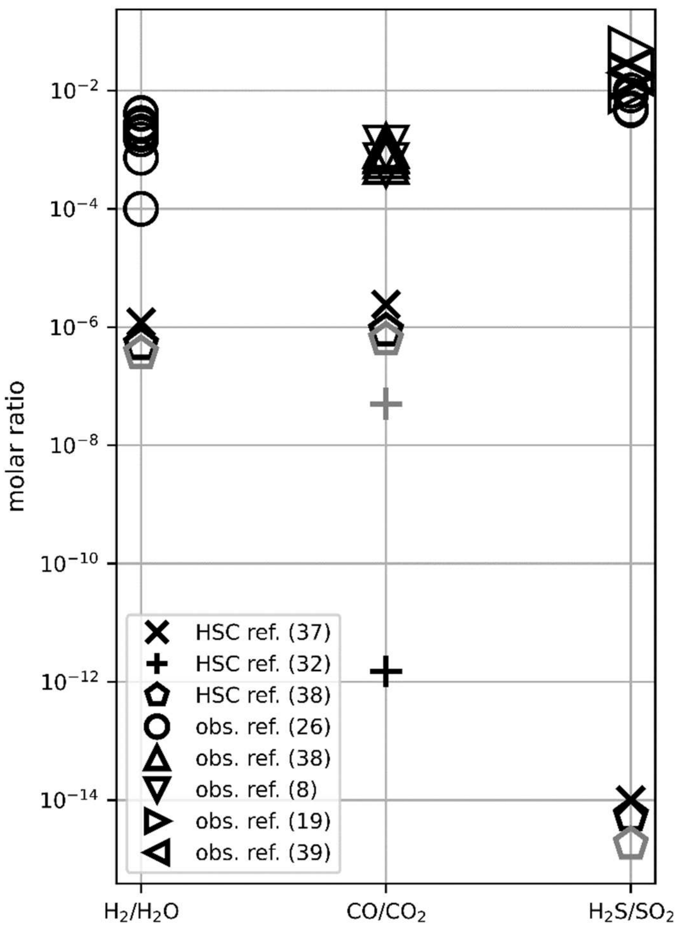

Fig. S 1: Comparison between redox couples measured in Mt Etna plume and calculated by the thermochemical equilibrium model HSC. Mt Etna plume measurements are by in-situ electrochemical sensor (H_2/H_2O, H_2S/SO_2) during passive degassing, or FTIR remote sensing of lava fountain events. HSC model calculation from *(37)* corresponds to an ArcMean gas composition *(36)*, at $T = 1273$ K and atmospheric to magmatic gas volume mixing ratio of $V_A/V_M = 0.1$. Calculations from *(32)* are based on gas composition for two scenarios: (1) Mt Etna: $V_A{:}V_M = 40{:}60$, $T = 873$ K (black marker) and (2) ArcMean: $V_A{:}V_M = 15{:}85$, $T = 1173$ K (grey marker). Calculations of *(38)* represent the plume of Masaya volcano at one temperature of $T = 1273$ K and two different mixing conditions: $V_A{:}V_M = 5{:}95$ (black marker) and $V_A{:}V_M = 10{:}90$ (grey marker).

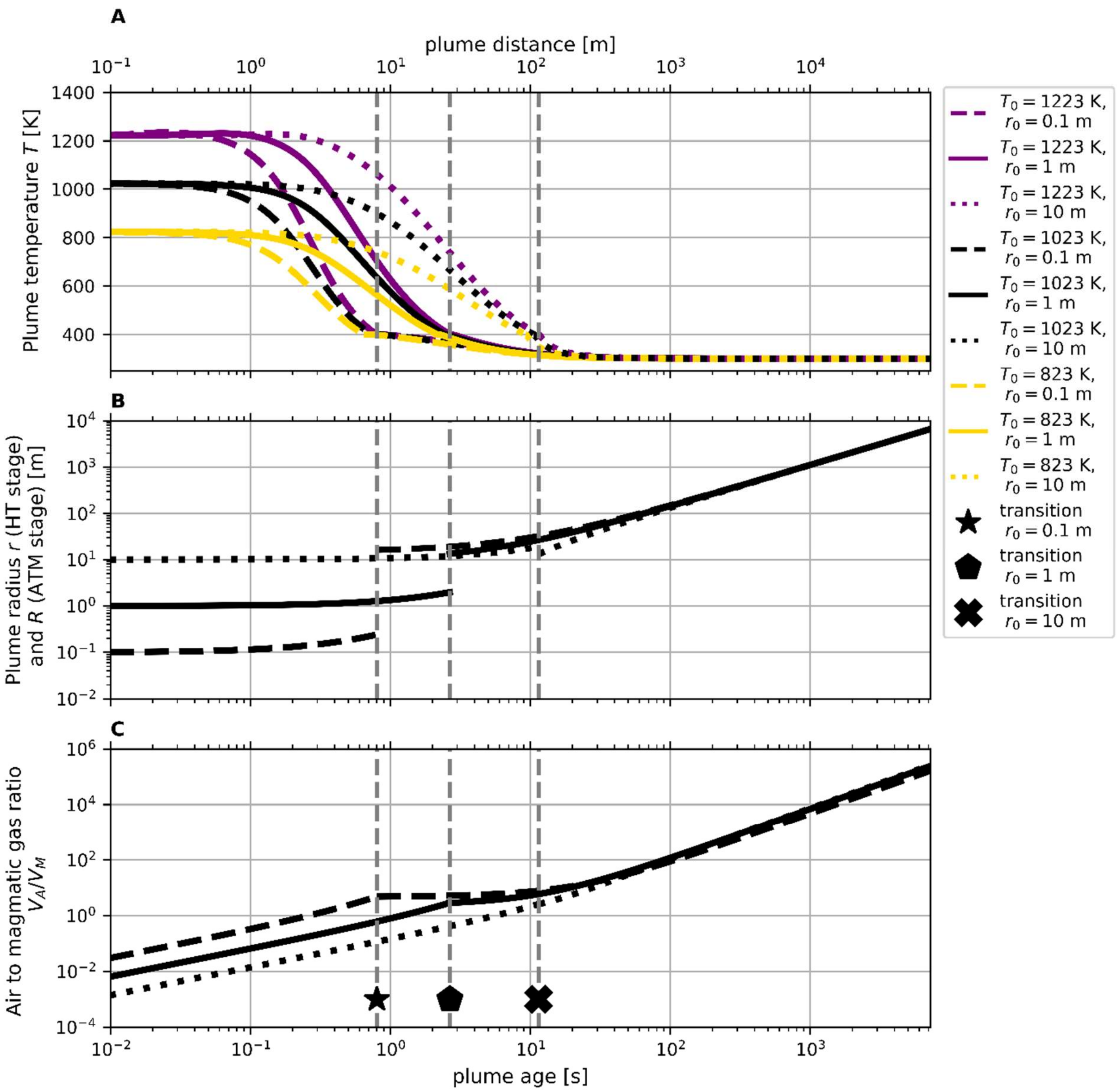

Fig. S 2: **Plume physical parameters for the intermediate emission temperatures in Fig. 2.** Physical parameters of the modeled turbulent mixing scheme as a function of plume evolution. Plume age (lower x-axis, in [s]) is also shown as equivalent distance for an assumed wind speed of 10 m s^{-1} (upper x-axis, in [m]). Nine simulations are presented with different magmatic gas temperatures (T_0 = 1223 (purple lines), 1023 (black lines), 823 (gold lines) K) and magmatic gas bubble radii/mixing scenarios (r_0 = 0.1, 1, 10 m). The black markers and the grey vertical lines show the 400 K transition region between the HT and the ATM stage of the model. Panel A shows the plume temperature evolution. Panel B shows the evolution of the plume radii, *r* (HT plume) and *R* (ATM plume). Panel C shows the evolution of the ratio between in-mixed atmospheric air volume V_A to the initial volume of magmatic gas V_M. Since HT plume mixing depends only on the initial source radius, r_0, i.e. the radius of the emitted magmatic gas bubbles (and not temperature), panel B and C show only one line per bubble radius.

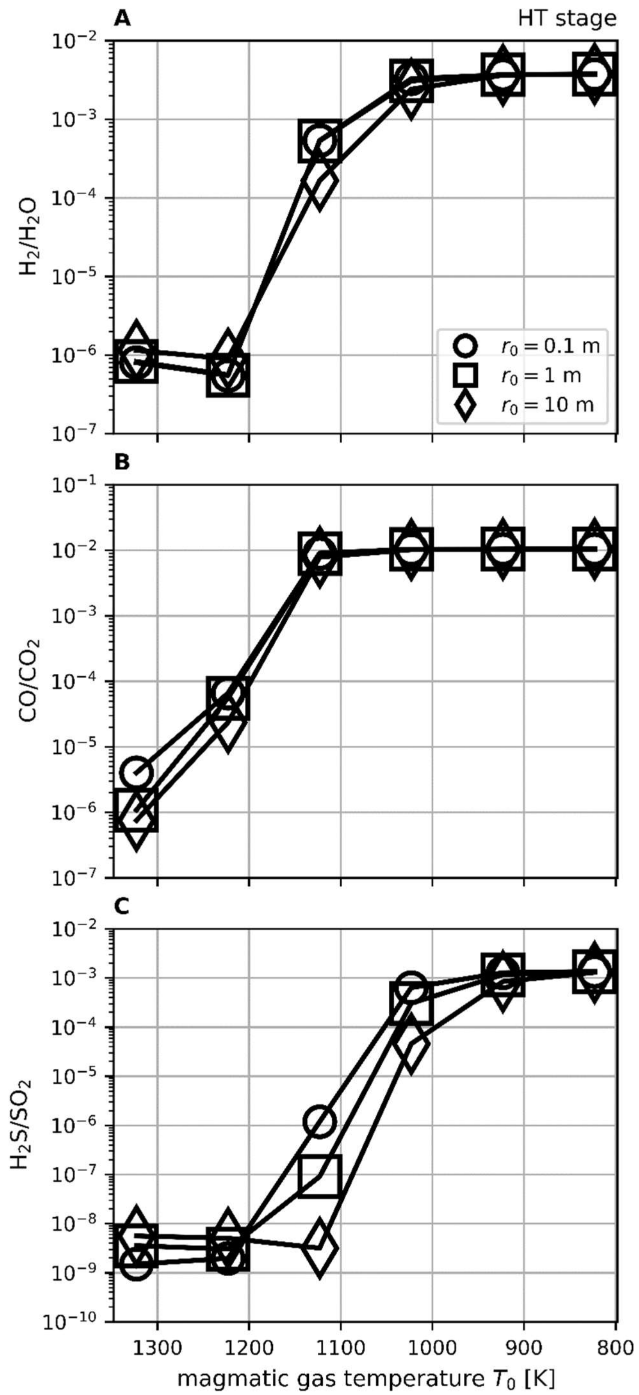

Fig. S 3: **Redox pairs after HT stage oxidative plume processing for three different mixing scenarios.** Molar ratios of the three most relevant redox couples (H_2/H_2O (panel A), CO/CO_2 (panel B), H_2S/SO_2 (panel C)) in the volcanic plume displayed at the end of the HT model stage (T_{plume} = 400 K) after all possible rapid high-temperature oxidation processes have terminated. Simulations are performed across a range of magmatic gas temperatures T_0 corresponding to different amounts of magmatic gas cooling prior to emission to air. The different markers show the model yields very similar results for the three mixing scenarios with source radii (emitted magmatic gas bubble radii) of r_0 = 0.1, 1, 10 m.

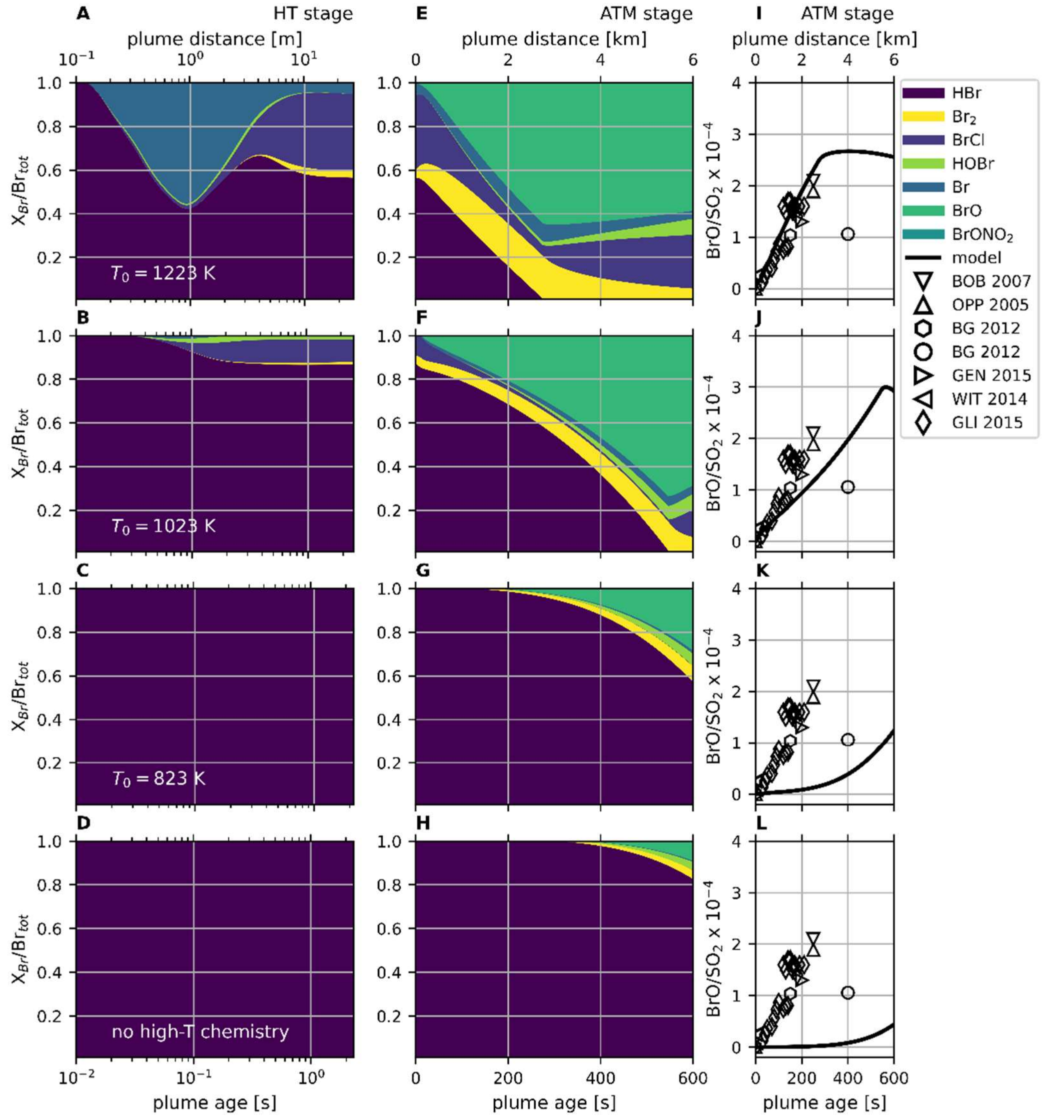

Fig. S 4: **Overview of relative bromine speciation and BrO/SO2 ratios simulated across HT and ATM stages for intermediate temperatures.** Relative bromine speciation and BrO/SO2 for the remaining magmatic gas temperature runs (T_0 = 1223, 1023, 823 K) compared to Fig. 4, demonstrating very similar results. Overview of relative bromine speciation, X_{Br}/Br_{tot} and BrO/SO2 ratios simulated across HT and ATM stages of the model as a function of time (lower x-axis, [s]) and equivalent distance (upper x-axis, [m], or [km]). The different rows correspond to different magmatic gas temperatures (row A, E, I: T_0 = 1223 K, row B, F, J: T_0 = 1023 K, row C, G, K: T_0 = 823 K, row D, H, L: reference run without high temperature chemistry in HT stage). The first column (panel A – D) shows the evolution in relative bromine speciation during the HT plume stage through cooling to 400 K. Note the logarithmic time axis. The second column (panel E – H) shows the relative bromine speciation during the ATM stage (which has been initialized with output from the HT stage), during the first 10 min of plume evolution (with linear time axis). The last column (panel I – L) shows the BrO/SO2 ratio for these simulation runs (black lines) compared to near source (distance < 6 km, equivalent to < 600 s for a 10 m s^{-1} wind speed) plume observations (open markers) from the compilation of *(42)*.

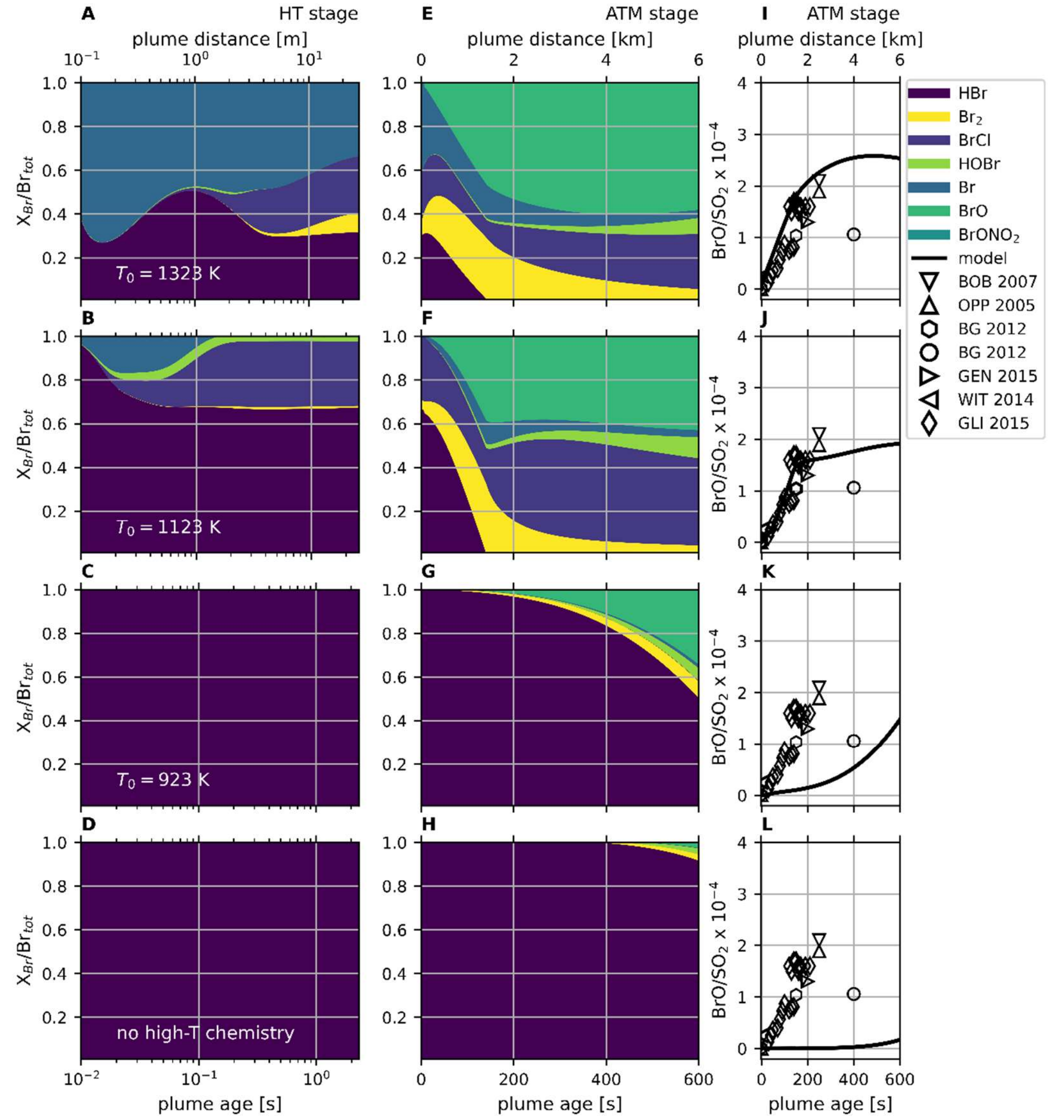

Fig. S 5: **Overview of relative bromine speciation and BrO/SO$_2$ ratios simulated across HT and ATM stages for a small magmatic gas bubble radius.** Relative bromine speciation and BrO/SO$_2$ for a magmatic gas bubble radius of $r_0 = 0.1$ m, demonstrating very similar results to Fig. 4. Overview of relative bromine speciation, X_{Br}/Br_{tot} and BrO/SO$_2$ ratios simulated across HT and ATM stages of the model as a function of time (lower x-axis, [s]) and equivalent distance (upper x-axis, [m], or [km]). The different rows correspond to different magmatic gas temperatures (row A, E, I: $T_0 = 1323$ K, row B, F, J: $T_0 = 1123$ K, row C, G, K: $T_0 = 923$ K, row D, H, L: reference run without high temperature chemistry in HT stage). The first column (panel A – D) shows the evolution in relative bromine speciation during the HT plume stage through cooling to 400 K. Note logarithmic time axis. The second column (panel E – H) shows the relative bromine speciation during the ATM stage (which has been initialized with output from the HT stage), during the first 10 min of plume evolution (with linear time axis). The last column (panel I – L) shows the BrO/SO$_2$ ratio for these simulation runs (black lines) compared to near source (distance < 6 km, equivalent to < 600 s for a 10 m s^{-1} wind speed) plume observations (open markers) from the compilation of *(42)*.

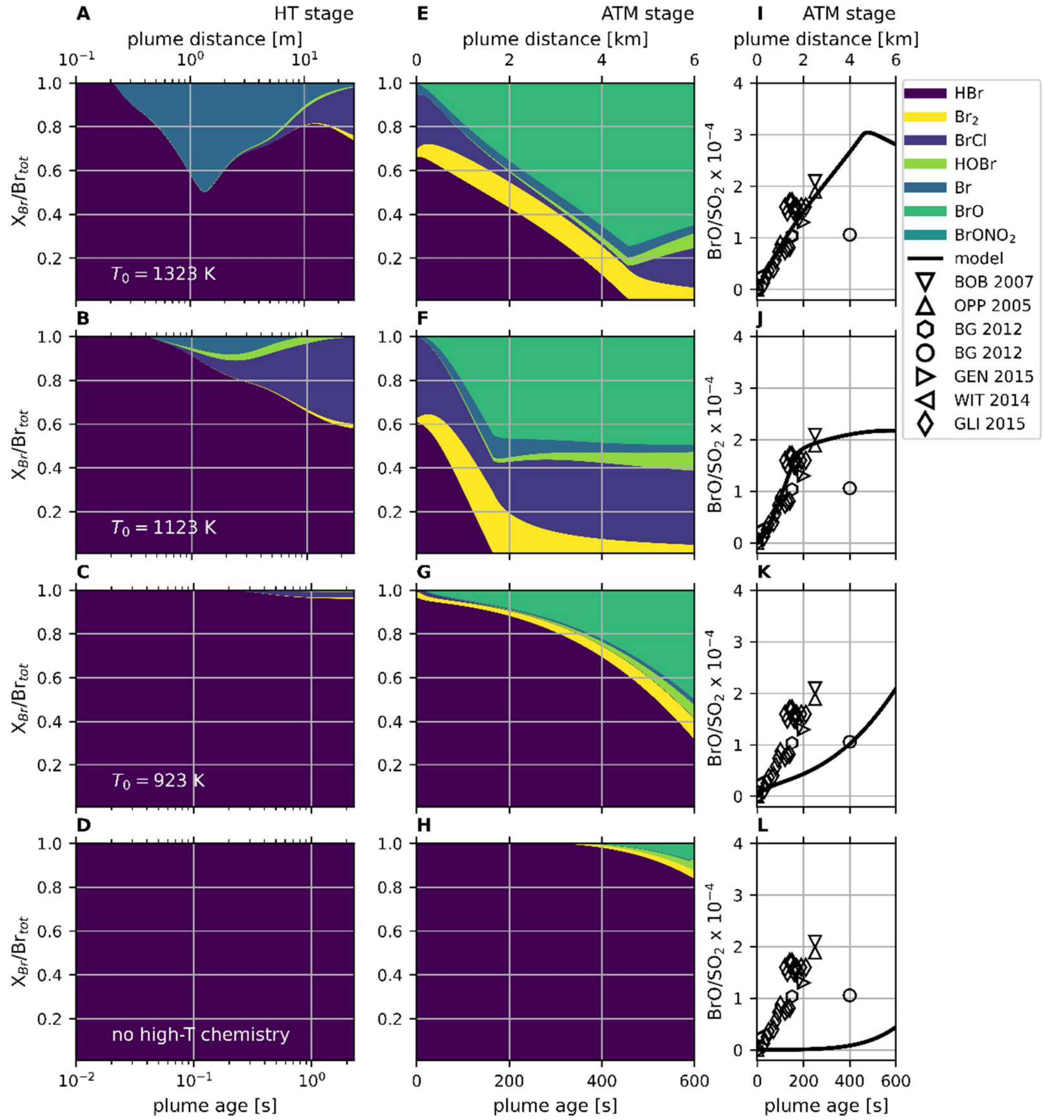

Fig. S 6: **Overview of relative bromine speciation and BrO/SO_2 ratios simulated across HT and ATM stages for a large magmatic gas bubble radius.** Relative bromine speciation and BrO/SO_2 for a magmatic gas bubble radius of $r_0 = 10$ m, demonstrating very similar results to Fig. 4. Overview of relative bromine speciation, X_{Br}/Br_{tot} and BrO/SO_2 ratios simulated across HT and ATM stages of the model as a function of time (lower x-axis, [s]) and equivalent distance (upper x-axis, [m], or [km]). The different rows correspond to magmatic gas temperatures (row A, E, I: T_0 = 1323 K, row B, F, J: T_0 = 1123 K, row C, G, K: T_0 = 923 K, row D, H, L: reference run without high temperature chemistry in HT stage). The first column (panel A – D) shows the evolution in relative bromine speciation during the HT plume stage through cooling to 400 K. Note logarithmic time axis. The second column (panel E – H) shows the relative bromine speciation during the ATM stage (which has been initialized with output from the HT stage), during the first 10 min of plume evolution (with linear time axis). The last column (panel I – L) shows the BrO/SO_2 ratio for these simulation runs (black lines) compared to near source (distance < 6 km, equivalent to < 600 s for a 10 m s^{-1} wind speed) plume observations (open markers) from the compilation of *(42)*.

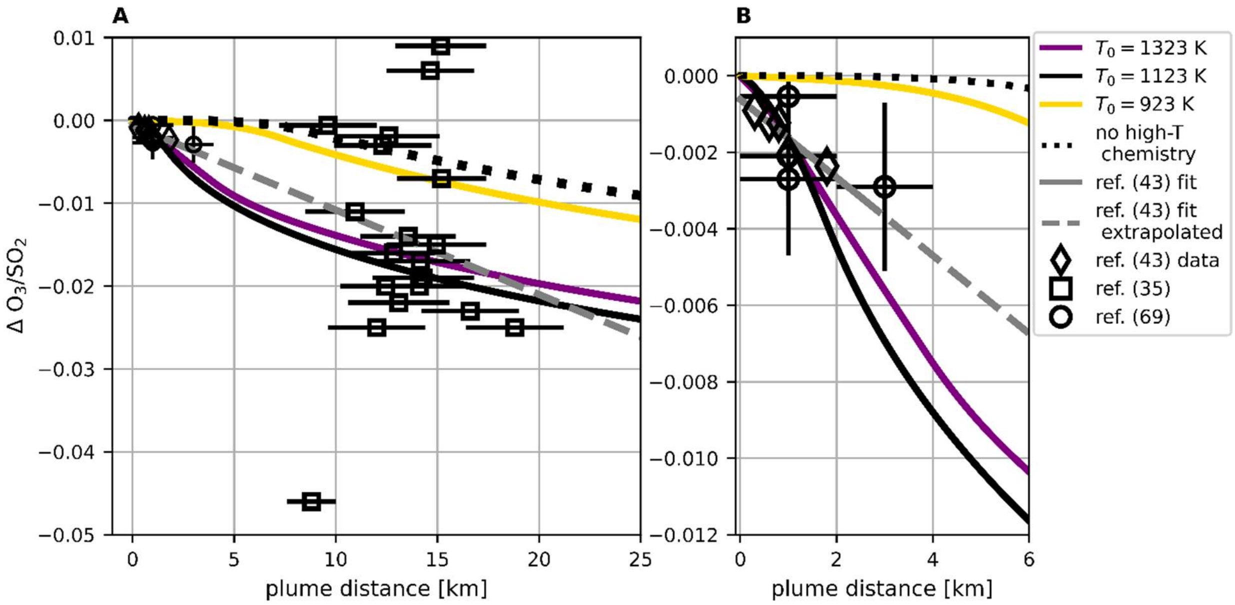

Fig. S 7: Ozone loss in the plume through BrO formation. Ozone loss in the plume for simulations with three magmatic gas temperatures at T_0 = 1323 K (purple line), 1123 K (black line), and 923 K (yellow line), as well as a control run without any high-temperature chemistry (dotted line). All model runs use a magmatic bubble radius of r_0 = 1 m . The O_3 destruction is shown as $\Delta O_3/SO_2$ ratio, where ΔO_3 is the difference between modelled in-plume O_3 and background O_3 and SO_2 is effectively a plume tracer thus corrects for plume dilution. Panel A shows the complete dataset and panel B a zoom over the first 6 km (600s) downwind. The simulated ozone loss is compared to reported in-plume observations on different platforms: ground-base *(43)*, aircraft-based *(35)* and drone-based *(69)*. The observations show considerable scatter but confirm ozone loss in the downwind plume at magnitudes predicted by the model for the two higher magmatic gas temperatures. The modeled ozone loss is caused by BrO chemistry (e.g. reaction cycle R1a, R1b, R2a, see Table S 2). The simulations for an emission at 923 K and control run without high-temperature chemistry both underestimate ozone loss, consistent with the underestimated BrO in these plumes due to inefficient (or absent) generation of early plume reactive bromine by high-temperature oxidative processing.

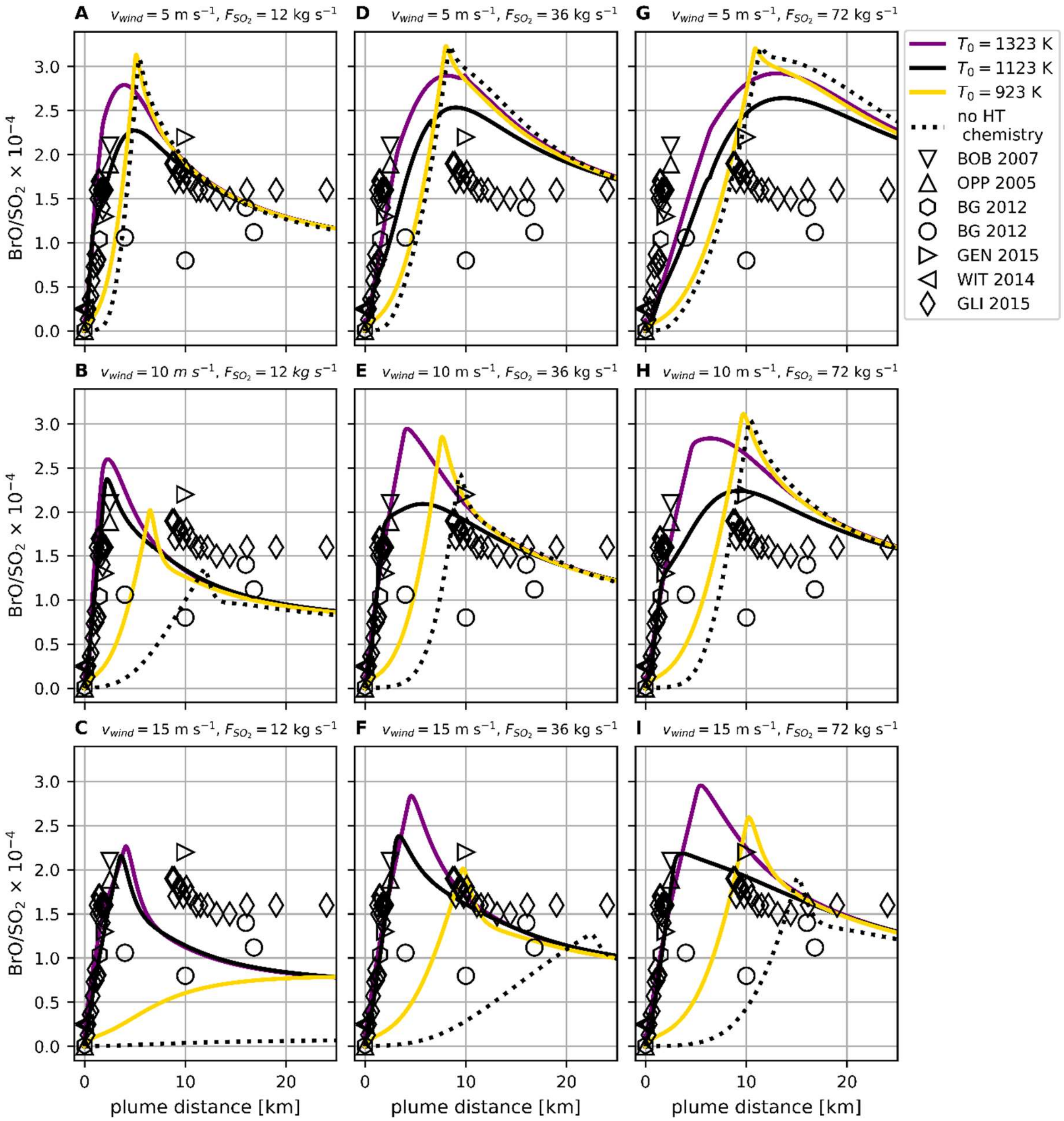

Fig. S 8: Impact of varying atmospheric mixing on BrO/SO$_2$ in the downwind plume. Simulated BrO/SO$_2$ in Mt. Etna plume as a function of distance downwind for three different magmatic gas temperatures (T_0 = 1323 K (purple line), 1123 K (black line), 923 K (yellow line), and control run with no high-temperature chemistry, dotted line) and for nine different atmospheric mixing scenarios that combine three SO$_2$ fluxes (columns left to right: F_{SO_2} = 12 kg s^{-1} (panel A-C), 36 kg s^{-1} (panel D-F), 72 kg s^{-1} (panel G-I)), and three wind speeds (upper row: v_{wind} = 5 m s^{-1} (panel A, D, G), middle row: 10 m s^{-1} (panel B, E, H), lower row: 15 m s^{-1} (panel C, F, I)). Model simulations are compared to observational data compiled by *(42)* (black open markers). This sensitivity study shows that the initial rise in BrO/SO$_2$ in the near-downwind plume (< 6 km downwind) is largely independent of the choice of atmospheric dispersion parameters (SO$_2$ flux and wind speed), and that gas emissions at low temperatures (923 K) or without high-temperature chemistry underestimate the observed rapid formation of BrO in the first few km downwind.

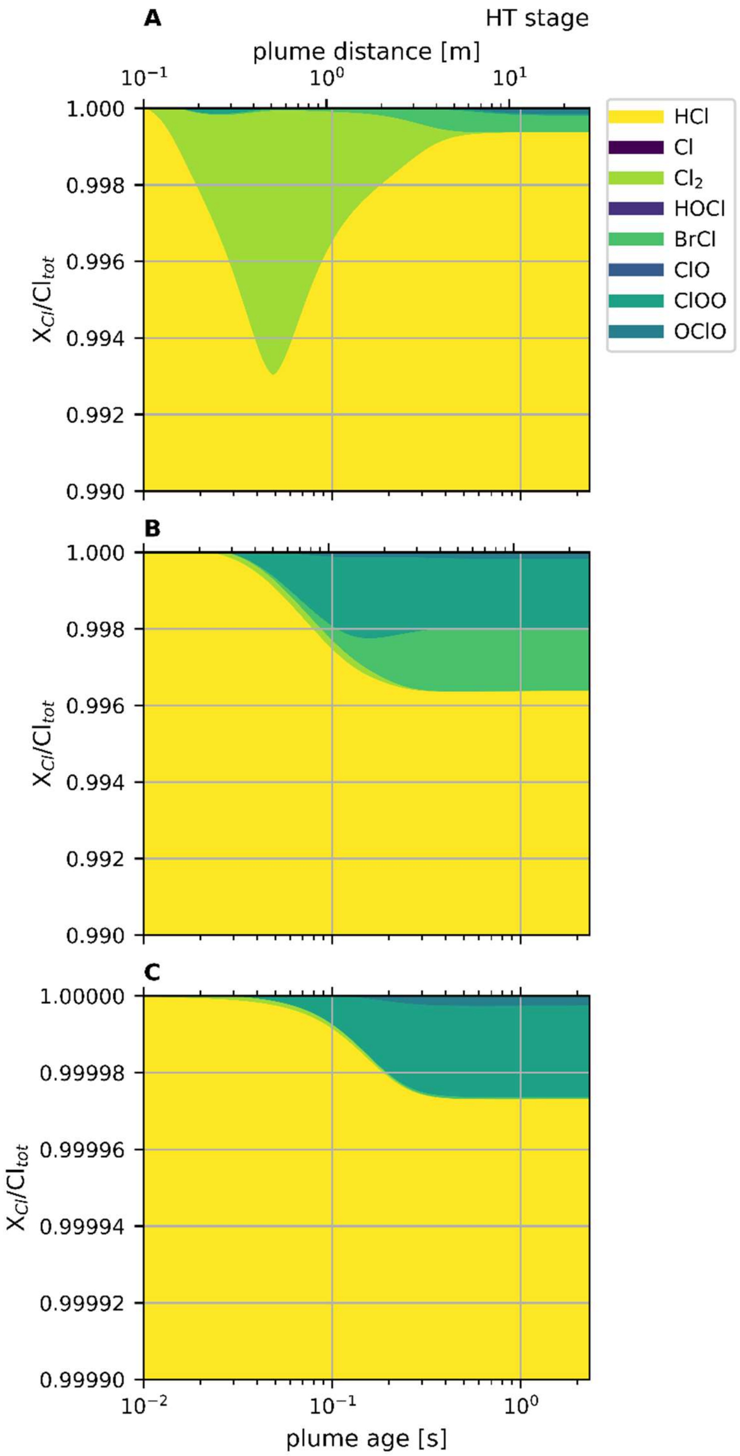

Fig. S 9: Chlorine speciation in Mt. Etna HT stage plume. Overview of relative chlorine speciation, X_{Cl}/Cl_{tot} in the HT-stage from emission through cooling to 400 K. Model simulations are for three different magmatic gas temperatures (A: T_0 = 1323 K, B: T_0 = 1123 K, C: T_0 = 923 K. Note logarithmic time axis, and truncated y-axis with the vast majority of chlorine remaining in the emitted form, HCl.

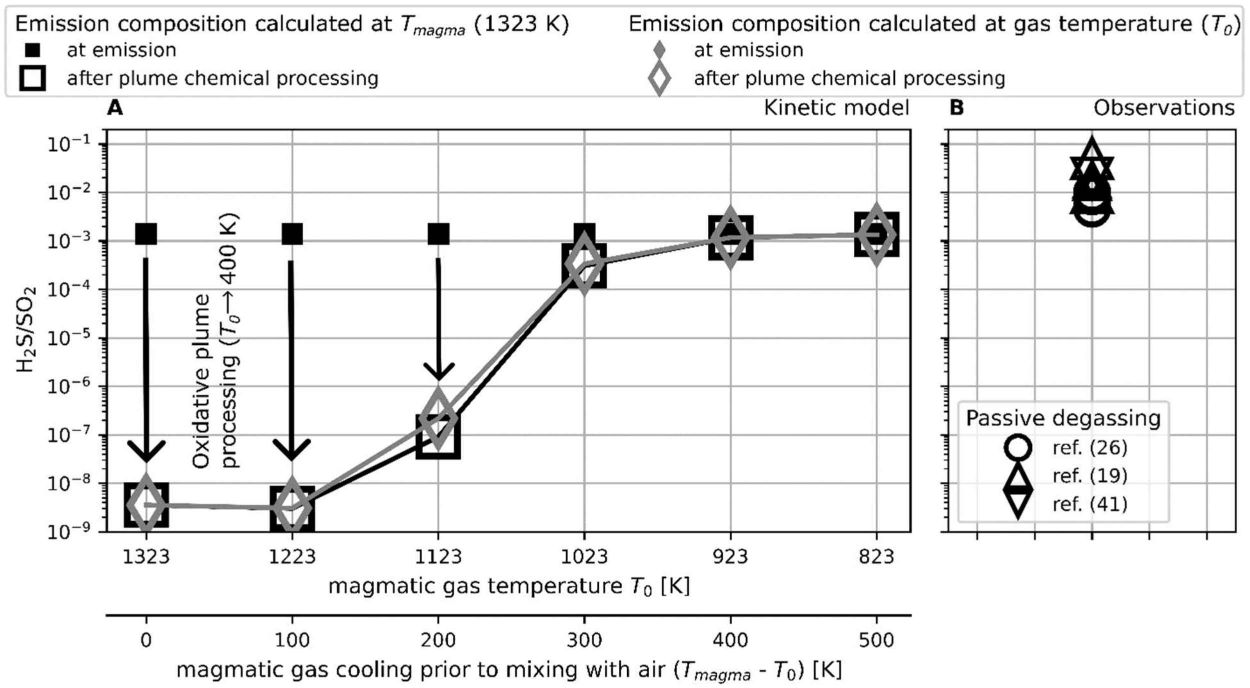

Fig. S 10: Evolution of H_2S/SO_2 ratio in HT stage compared to observations. Simulated H_2S/SO_2 ratio in the cooled plume (T_{plume} = 400 K, after all possible rapid high-temperature oxidation processes have terminated) compared to the ratio in model emission and plume observations. Simulations are performed across a range of magmatic gas temperatures T_0 corresponding to different amounts of magmatic gas cooling prior to emission to air. The left column shows the model results (open markers) compared to the initializations at emission (closed markers). Shown are two simulation variants: black markers correspond to simulation runs with a constant gas emission composition released at the magmatic gas temperature T_0 and grey markers show simulation runs where the emission composition of H_2/H_2O and CO/CO_2 are buffered by the NNO+0.35 redox buffer, and H_2S/SO_2 was held constant and not controlled by magma oxidation state following *(11)*. These model runs are only subtly different (black and gray open markers), and are identical in their starting H_2S/SO_2 in the emission (only shown with closed black markers). The right column shows field-observations at Mt. Etna which reflect a slightly more oxidized composition than the model emissions based on NNO+0.35. The model simulations show H_2S/SO_2 is impacted at higher gas emission temperatures, similar to CO and H_2. However, the complexity and uncertainties in H_2S combustion kinetics (for a review see *(47)*, as well as recent papers (e.g. *(46-49)*) on this active and evolving topic in combustion science) lends caution to detailed interpretation of the HT stage model simulations of H_2S/SO_2 at this time.

Reference	Reaction
1) Activation of halogen radicals	
1a	$HBr + OH \leftrightarrow Br + H_2O$
1b	$HCl + OH \leftrightarrow Cl + H_2O$
2) Further speciation of Bromine species	
2a	$Br + HO_2 \leftrightarrow BrO + OH$
2b	$BrO + H_2O \leftrightarrow HOBr + OH$
2c	$BrO + HBr \leftrightarrow HOBr + Br$
3) Further speciation of Chlorine species	
3a	$Cl + HO_2 \leftrightarrow ClO + OH$
3b	$ClO + HCl \leftrightarrow HOCl + Cl$
3c	$HOCl + Cl \leftrightarrow Cl_2 + OH$
3d	$Cl_2 + H \leftrightarrow HCl + Cl$
3e	$Cl + HO_2 \leftrightarrow HCl + O_2$
4) Interhalogen reactions	
4a	$Cl_2 + Br \leftrightarrow BrCl + Cl$
4b	$HOBr + Cl \leftrightarrow BrCl + OH$
4c	$HBr + Cl \leftrightarrow Br + HCl$
4d	$Br + BrCl \leftrightarrow Br_2 + Cl$

Table S 1: Key high-temperature reactions related to bromine and chlorine transformations in the HT model stage. Both forward and reverse reactions are simulated in the HT stage. High-temperature formation of OH in the HT stage volcanic plume drives the formation of halogen radicals (Br and Cl) in reaction R1a and R1b. Further speciation changes occur according to temperature evolution and mixing scenario of the HT stage plume.

Reference	Reaction
1) O_3 destruction by halogen radicals	
1a	$Br + O_3 \rightarrow BrO + O_2$
1b	$Cl + O_3 \rightarrow ClO + O_2$
2) Gas-phase recycling of BrO	
2a	$BrO + BrO \rightarrow Br_2 + O_2$
2b	BrO + BrO → 2 Br + O2
3) Heterogeneous recycling of BrO	
3a	$BrO + HO_2 \rightarrow HOBr + O_2$
3b	$HOBr + Br^-_{aq} + H^+_{aq} \rightarrow Br_2 + H_2O$
3c	$HOBr + Cl^-_{aq} + H^+_{aq} \rightarrow BrCl + H_2O$
4) Photochemical activation of halogen radicals	
4a	$Br_2 + h\nu \rightarrow 2\,Br$
4b	$BrCl + h\nu \rightarrow Br + Cl$

Table S 2: Key reactions related to the destruction of O_3 and the 'bromine explosion' mechanism in the ATM model stage. O_3 molecules mixed into the volcanic plume react in a first step with halogen radicals (Br and Cl) to form BrO and ClO, respectively (R1a, R1b). BrO can be recycled via gas-phase self-reactions (R2a, R2b) and a heterogeneous pathway involving the reactive uptake of HOBr on acidic aerosols containing dissolved halogen halides HBr and HCl (R3a-R3c). The product of these two BrO recycling pathways is either Br_2 or BrCl, which undergoes photochemistry and recovers the initial halogen radicals (R4a, R4b). Under typical volcanic emission compositions, aqueous-phase equilibria drive towards the Br_2 product, thereby converting Br^-_{aq} (hence HBr) into reactive bromine. This starts an autocatalytic reaction scheme called the 'bromine explosion' *(31)*.

REFERENCES AND NOTES

1. W. F. Giggenbach, Geothermal gas equilibria. *Geochim. Cosmochim. Acta* **44**, 2021–2032 (1980).

2. R. Scarpa, R. I. Tilling, *Monitoring and Mitigation of Volcano Hazards* (Springer Berlin Heidelberg, 1996).

3. T. P. Fischer, W. F. Giggenbach, Y. Sano, S. N. Williams, Fluxes and sources of volatiles discharged from Kudryavy, a subduction zone volcano, Kurile Islands. *Earth Planet. Sci. Lett.* **160**, 81–96 (1998).

4. Yu. A. Taran, J. W. Hedenquist, M. A. Korzhinsky, S. I. Tkachenko, K. I. Shmulovich, Geochemistry of magmatic gases from Kudryavy volcano, Iturup, Kuril Islands. *Geochim. Cosmochim. Acta* **59**, 1749–1761 (1995).

5. I. V. Chaplygin, V. Y. Lavrushin, E. O. Dubinina, Y. V. Bychkova, S. Inguaggiato, M. A. Yudovskaya, Geochemistry of volcanic gas at the 2012–13 New Tolbachik eruption, Kamchatka. *J. Volcanol. Geotherm. Res.* **323**, 186–193 (2016).

6. W. F. Giggenbach, D. Tedesco, Y. Sulistiyo, A. Caprai, R. Cioni, R. Favara, T. P. Fischer, J.-I. Hirabayashi, M. Korzhinsky, M. Martini, I. Menyailov, H. Shinohara, Evaluation of results from the fourth and fifth IAVCEI field workshops on volcanic gases, Vulcano island, Italy and Java, Indonesia. *J. Volcanol. Geotherm. Res.* **108**, 157–172 (2001).

7. Y. Moussallam, C. Oppenheimer, A. Aiuppa, G. Giudice, M. Moussallam, P. Kyle, Hydrogen emissions from Erebus volcano, Antarctica. *Bull. Volcanol.* **74**, 2109–2120 (2012).

8. P. Allard, M. Burton, F. Murè, Spectroscopic evidence for lava fountain driven by previously accumulated magmatic gas. *Nature* **433**, 407–410 (2005).

9. A. Aiuppa, Y. Moussallam, Hydrogen and hydrogen sulphide in volcanic gases: Abundance, processes, and atmospheric fluxes. *C. R. Géosci.* **356**, 85–108 (2024).

10. T. M. Gerlach, Oxygen buffering of Kilauea volcanic gases and the oxygen fugacity of Kilauea basalt. *Geochim. Cosmochim. Acta* **57**, 795–814 (1993).

11. W. F. Giggenbach, Redox processes governing the chemistry of fumarolic gas discharges from White Island, New Zealand. *Appl. Geochem.* **2**, 143–161 (1987).

12. R. W. Henley, T. P. Fischer, Sulfur sequestration and redox equilibria in volcanic gases. *J. Volcanol. Geotherm. Res.* **414**, 107181 (2021).

13. C. Oppenheimer, B. Scaillet, A. Woods, A. J. Sutton, T. Elias, Y. Moussallam, Influence of eruptive style on volcanic gas emission chemistry and temperature. *Nat. Geosci.* **11**, 678–681 (2018).

14. Y. Moussallam, C. Oppenheimer, B. Scaillet, A novel approach to volcano surveillance using gas geochemistry. *C. R. Géosci.* **356**, 71–84 (2022).

15. Y. Moussallam, C. Oppenheimer, B. Scaillet, On the relationship between oxidation state and temperature of volcanic gas emissions. *Earth Planet. Sci. Lett.* **520**, 260–267 (2019).

16. R. Atkinson, D. L. Baulch, R. A. Cox, J. N. Crowley, R. F. Hampson, R. G. Hynes, M. E. Jenkin, M. J. Rossi, J. Troe, Evaluated kinetic and photochemical data for atmospheric chemistry: Volume I—Gas phase reactions of O_x, HO_x, NO_x and SO_x species. *Atmos. Chem. Phys.* **4**, 1461–1738 (2004).

17. J. B. Burkholder, S. P. Sander, J. Abatt, J. R. Barker, C. Cappa, J. D. Crounse, T. S. Dibble, R. E. Huie, C. E. Kolb, M. J. Korylo, V. L. Orkin, C. J. Percival, D. M. Wilmouth, P. H. Wine, JPL Publication 19-5. Chemical Kinetics and Photochemical Data for Use in Atmospheric Studies (JPL Publications, 2019), vol. **19**.

18. J. A. Manion, R. E. Huie, R. D. Levin, D. R. Burgess Jr., V. L. Orkin, W. Tsang, W. S. McGivern, J. W. Hudgens, V. D. Knyazev, D. B. Atkinson, E. Chai, A. M. Tereza, C. Y. Lin, T. C. Allison, W. G. Mallard, F. Westley, J. T. Herron, R. F. Hampson, D. H. Frizzel, NIST Chemical Kinetics Database, NIST Standard Reference Database 17, Version 7.0 (Web Version), version 2015.09, Release 1.6.8 (NIST, 2015); https://kinetics.nist.gov/.

19. A. Aiuppa, A. Franco, M. Valenza, The tropospheric processing of acidic gases and hydrogen sulphide in volcanic gas plumes as inferred from field and model investigations. *Atmos. Chem. Phys.* **7**, 1441–1450 (2007).

20. J. Kuhn, N. Bobrowski, U. Platt, The interface between magma and Earth's atmosphere. *Geochem. Geophys. Geosyst.* **23**, e2022GC010671 (2022).

21. R. S. Martin, E. Ilyinskaya, C. Oppenheimer, The enigma of reactive nitrogen in volcanic emissions. *Geochim. Cosmochim. Acta* **95**, 93–105 (2012).

22. T. J. Roberts, G. Dayma, C. Oppenheimer, Reaction rates control high-temperature chemistry of volcanic gases in air. *Front. Earth Sci.* **7**, 154 (2019).

23. R. S. Martin, T. J. Roberts, T. A. Mather, D. M. Pyle, The implications of H_2S and H_2 kinetic stability in high-T mixtures of magmatic and atmospheric gases for the production of oxidized trace species (e.g., BrO and NO_x). *Chem. Geol.* **263**, 143–150 (2009).

24. D. P. Cruikshank, D. Morrison, K. Lennon, Volcanic gases: Hydrogen burning at Kilauea Volcano, Hawaii. *Science* **182**, 277–279 (1973).

25. R. Kazahaya, M. Varnam, B. Esse, M. Burton, H. Shinohara, M. Ibarra, Behaviors of redox-sensitive components in the volcanic plume at Masaya volcano, Nicaragua: H_2 oxidation and CO preservation in air. *Front. Earth Sci.* **10**, 867562 (2022).

26. A. Aiuppa, H. Shinohara, G. Tamburello, G. Giudice, M. Liuzzo, R. Moretti, Hydrogen in the gas plume of an open-vent volcano, Mount Etna, Italy. *J. Geophys. Res. Solid Earth* **116**, B10204 (2011).

27. J. Kuhn, N. Bobrowski, G. Boudoire, S. Calabrese, G. Giuffrida, M. Liuzzo, K. Karume, D. Tedesco, T. Wagner, U. Platt, High-spectral-resolution Fabry-Pérot interferometers overcome fundamental limitations of present volcanic gas remote sensing techniques. *Front. Earth Sci.* **11**, 1039093 (2023).

28. N. Bobrowski, G. Hönninger, B. Galle, U. Platt, Detection of bromine monoxide in a volcanic plume. *Nature* **423**, 273–276 (2003).

29. N. Bobrowski, G. Giuffrida, Bromine monoxide/sulphur dioxide ratios in relation to volcanological observations at Mt. Etna 2006-2009. *Solid Earth* **3**, 433–445 (2012).

30. J. Gliß, N. Bobrowski, L. Vogel, D. Pöhler, U. Platt, OClO and BrO observations in the volcanic plume of Mt. Etna—Implications on the chemistry of chlorine and bromine species in volcanic plumes. *Atmos. Chem. Phys.* **15**, 5659–5681 (2015).

31. L. Barrie, U. Platt, Arctic tropospheric chemistry: An overview. *Tellus B Chem. Phys. Meteorol.* **49**, 450 (1997).

32. N. Bobrowski, R. von Glasow, A. Aiuppa, S. Inguaggiato, I. Louban, O. W. Ibrahim, U. Platt, Reactive halogen chemistry in volcanic plumes. *J. Geophys. Res. Atmos.* **112**, D06311 (2007).

33. H. Narivelo, P. D. Hamer, V. Marécal, L. Surl, T. Roberts, S. Pelletier, B. Josse, J. Guth, M. Bacles, S. Warnach, T. Wagner, S. Corradini, G. Salerno, L. Guerrieri, A regional modelling study of halogen chemistry within a volcanic plume of Mt Etna's Christmas 2018 eruption. *Atmos. Chem. Phys.* **23**, 10533–10561 (2023).

34. T. J. Roberts, R. S. Martin, L. Jourdain, Reactive bromine chemistry in Mount Etna's volcanic plume: The influence of total Br, high-temperature processing, aerosol loading and plume–air mixing. *Atmos. Chem. Phys.* **14**, 11201–11219 (2014).

35. L. Surl, T. Roberts, S. Bekki, Observation and modelling of ozone-destructive halogen chemistry in a passively degassing volcanic plume. *Atmos. Chem. Phys.* **21**, 12413–12441 (2021).

36. T. M. Gerlach, Volcanic sources of tropospheric ozone-depleting trace gases. *Geochem. Geophys. Geosyst.* **5**, Q09007 (2004).

37. R. S. Martin, T. A. Mather, D. M. Pyle, High-temperature mixtures of magmatic and atmospheric gases. *Geochem. Geophys. Geosyst.* **7**, Q04006 (2006).

38. J. Rüdiger, A. Gutmann, N. Bobrowski, M. Liotta, J. M. de Moor, R. Sander, F. Dinger, J.-L. Tirpitz, M. Ibarra, A. Saballos, M. Martínez, E. Mendoza, A. Ferrufino, J. Stix, J. Valdés, J. M. Castro, T. Hoffmann, Halogen activation in the plume of Masaya volcano: Field observations and box model investigations. *Atmos. Chem. Phys.* **21**, 3371–3393 (2021).

39. R. B. Symonds, M. H. Reed, Calculation of multicomponent chemical-equilibria in gas-solid-liquid systems: Calculation methods, thermochemical data, and applications to studies of high temperature volcanic gases with examples from Mt. St. Helens. *Am. J. Sci.* **293**, 798–864 (1993).

40. A. La Spina, M. Burton, P. Allard, S. Alparone, F. Muré, Open-path FTIR spectroscopy of magma degassing processes during eight lava fountains on Mount Etna. *Earth Planet. Sci. Lett.* **413**, 123–134 (2015).

41. T. J. Roberts, T. Lurton, G. Giudice, M. Liuzzo, A. Aiuppa, M. Coltelli, D. Vignelles, G. Salerno, B. Couté, M. Chartier, R. Baron, J. R. Saffell, B. Scaillet, Validation of a novel Multi-Gas sensor for volcanic HCl alongside H_2S and SO_2 at Mt. Etna. *Bull. Volcanol.* **79**, 36 (2017).

42. A. Gutmann, N. Bobrowski, T. J. Roberts, J. Rüdiger, T. Hoffmann, Advances in bromine speciation in volcanic plumes. *Front. Earth Sci.* **6**, 213 (2018).

43. L. Surl, D. Donohoue, A. Aiuppa, N. Bobrowski, R. von Glasow, Quantification of the depletion of ozone in the plume of Mount Etna. *Atmos. Chem. Phys.* **15**, 2613–2628 (2015).

44. E. Lehrer, G. Hönninger, U. Platt, A one dimensional model study of the mechanism of halogen liberation and vertical transport in the polar troposphere. *Atmos. Chem. Phys.* **4**, 2427–2440 (2004).

45. W. F. Giggenbach, "Chemical composition of volcanic gases" in *Monitoring and Mitigation of Volcano Hazards* (Springer Berlin Heidelberg, 1996), pp. 221–256.

46. J. M. Colom-Díaz, Á. Millera, R. Bilbao, M. U. Alzueta, New results of H_2S oxidation at high pressures. Experiments and kinetic modeling. *Fuel* **285**, 119261 (2021).

47. A. Elkhazraji, Q. Wang, M. Monge-Palacios, J. Zou, A. Alshaarawi, A. C. Sepulveda, S. M. Sarathy, A. Farooq, Oxidation of hydrogen sulfide and CO_2 mixtures: Laser-based multi-speciation and kinetic modeling. *Chem. Eng. J.* **486**, 150421 (2024).

48. M. Monge-Palacios, Q. Wang, A. Alshaarawi, A. C. Cavazos Sepulveda, S. M. Sarathy, Quantum chemistry and kinetics of hydrogen sulphide oxidation. *Phys. Chem. Chem. Phys.* **26**, 3219–3228 (2024).

49. A. Raj, S. Ibrahim, A. Jagannath, Combustion kinetics of H_2S and other sulfurous species with relevance to industrial processes. *Prog. Energy Combust. Sci.* **80**, 100848 (2020).

50. R. A. Daly, The nature of volcanic action. *Proc. Am. Acad. Arts Sci.* **47**, 47–122 (1911).

51. J. M. de Moor, T. P. Fischer, Z. D. Sharp, P. L. King, M. Wilke, R. E. Botcharnikov, E. Cottrell, M. Zelenski, B. Marty, K. Klimm, C. Rivard, D. Ayalew, C. Ramirez, K. A. Kelley, Sulfur degassing at Erta Ale (Ethiopia) and Masaya (Nicaragua) volcanoes: Implications for degassing processes and oxygen fugacities of basaltic systems. *Geochem. Geophys. Geosyst.* **14**, 4076–4108 (2013).

52. S. Hidalgo, J. Battaglia, S. Arellano, D. Sierra, B. Bernard, R. Parra, P. Kelly, F. Dinger, C. Barrington, P. Samaniego, Evolution of the 2015 Cotopaxi eruption revealed by combined geochemical and seismic observations. *Geochem. Geophys. Geosyst.* **19**, 2087–2108 (2018).

53. N. M. Abumounshar, S. Ibrahim, A. Raj, A detailed reaction mechanism for elemental sulphur combustion in the furnace of sulphuric acid plants. *Can. J. Chem. Eng.* **99**, 2441–2451 (2021).

54. B. Galle, M. Johansson, C. Rivera, Y. Zhang, M. Kihlman, C. Kern, T. Lehmann, U. Platt, S. Arellano, S. Hidalgo, Network for Observation of Volcanic and Atmospheric Change (NOVAC)—A global network for volcanic gas monitoring: Network layout and instrument description. *J. Geophys. Res. Atmos.* **115**, D05304 (2010).

55. F. Dinger, T. Kleinbek, S. Dörner, N. Bobrowski, U. Platt, T. Wagner, M. Ibarra, E. Espinoza, SO_2 and BrO emissions of Masaya volcano from 2014 to 2020. *Atmos. Chem. Phys.* **21**, 9367–9404 (2021).

56. S. Warnach, H. Sihler, C. Borger, N. Bobrowski, S. Beirle, U. Platt, T. Wagner, A new accurate retrieval algorithm of bromine monoxide columns inside minor volcanic plumes from Sentinel-5P TROPOMI observations. *Atmos. Meas. Tech.* **16**, 5537–5573 (2023).

57. Z. Zeng, B. Z. Dlugogorski, I. Oluwoye, M. Altarawneh, Combustion chemistry of COS and occurrence of intersystem crossing. *Fuel* **283**, 119257 (2021).

58. K. J. Hughes, L. Ma, R. T. J. Porter, M. Pourkashanian, "Mercury transformation modelling with bromine addition in coal derived flue gases" in *Computer Aided Chemical Engineering* (Elsevier, 2011), vol. **29**, pp. 171–175.

59. Y. Bedjanian, Experimental study of the reaction of $O(^3P)$ with carbonyl sulfide between 220 and 960 K. *J. Phys. Chem. A* **126**, 4080–4086 (2022).

60. Y. Bedjanian, Rate constant of the reaction of OH radicals with HBr over the temperature range 235–960 K. *J. Phys. Chem. A* **125**, 1754–1759 (2021).

61. L. Jourdain, T. J. Roberts, M. Pirre, B. Josse, Modeling the reactive halogen plume from Ambrym and its impact on the troposphere with the CCATT-BRAMS mesoscale model. *Atmos. Chem. Phys.* **16**, 12099–12125 (2016).

62. ACOM, Quick TUV (2024); https://acom.ucar.edu/Models/TUV/Interactive_TUV/.

63. E. Bagnato, A. Aiuppa, F. Parello, S. Calabrese, W. D'Alessandro, T. A. Mather, A. J. S. McGonigle, D. M. Pyle, I. Wängberg, Degassing of gaseous (elemental and reactive) and particulate mercury from Mount Etna volcano (Southern Italy). *Atmos. Environ.* **41**, 7377–7388 (2007).

64. A. Aiuppa, C. Federico, A. Franco, G. Giudice, S. Gurrieri, S. Inguaggiato, M. Liuzzo, A. J. S. McGonigle, M. Valenza, Emission of bromine and iodine from Mount Etna volcano. *Geochem. Geophys. Geosyst.* **6**, Q08008 (2005).

65. J. R. Holloway, V. Pan, G. Guðmundsson, High-pressure fluid-absent melting experiments in the presence of graphite: Oxygen fugacity, ferric/ferrous ratio and dissolved CO_2. *Eur. J. Mineral.* **4**, 105–114 (1992).

66. N. Metrich, R. Clocchiatti, Sulfur abundance and its speciation in oxidized alkaline melts. *Geochim. Cosmochim. Acta* **60**, 4151–4160 (1996).

67. T. J. Roberts, D. Vignelles, M. Liuzzo, G. Giudice, A. Aiuppa, M. Coltelli, G. Salerno, M. Chartier, B. Couté, G. Berthet, T. Lurton, F. Dulac, J.-B. Renard, The primary volcanic aerosol emission from Mt Etna: Size-resolved particles with SO_2 and role in plume reactive halogen chemistry. *Geochim. Cosmochim. Acta* **222**, 74–93 (2018).

68. F. A. Gifford, Tropospheric relative diffusion observations. *J. Appl. Meteorol.* **16**, 311–313 (1977).

69. M. Rüth, "Constructing a Miniaturized Chemiluminescence Ozone Monitor for Drone-Based Measurements in Volcanic Plumes: A Way to Resolve the Volcanic Ozone Enigma," thesis, Heidelberg University Library, Heidelberg, Germany (2024).
